# Chemisorption vs. Physisorption in Perfluorinated Zn(II) Porphyrin–SnO_2_ Hybrids for Acetone Chemoresistive Detection

**DOI:** 10.3390/molecules30244749

**Published:** 2025-12-12

**Authors:** Manuel Minnucci, Sara Oregioni, Eleonora Pargoletti, Gabriele Di Carlo, Francesca Tessore, Gian Luca Chiarello, Rocco Martinazzo, Mario Italo Trioni, Giuseppe Cappelletti

**Affiliations:** 1 Dipartimento di Chimica, Università degli Studi di Milano, Via Golgi 19, 20133 Milano, Italy; manuel.minnucci@unimi.it (M.M.); sara.oregioni@unimi.it (S.O.); eleonora.pargoletti@unimi.it (E.P.); gabriele.dicarlo@unimi.it (G.D.C.); gianluca.chiarello@unimi.it (G.L.C.); rocco.martinazzo@unimi.it (R.M.); giuseppe.cappelletti@unimi.it (G.C.); 2 Consorzio Interuniversitario Nazionale per la Scienza e Tecnologia dei Materiali (INSTM), Via Giusti 9, 50121 Firenze, Italy; 3 Institute of Chemical Sciences and Technologies “Giulio Natta”, National Research Council of Italy, Via Golgi 19, 20133 Milano, Italy; mario.trioni@scitec.cnr.it

**Keywords:** chemoresistors, gaseous acetone sensing, tin dioxide, porphyrins, nanocomposites, hybrids, density functional theory

## Abstract

In this study, the integration of SnO_2_ with a perfluorinated Zn(II) porphyrin derivative, namely ZnTPPF_20_CN, was explored as a strategy to enhance the performance of chemoresistive sensors toward gaseous acetone detection. The ZnTPPF_20_CN molecule was specifically designed with an ethynylphenyl-cyanoacrylic anchoring group and a benzothiadiazole (BTD) spacer, enabling its chemisorption onto the SnO_2_ surface. Hybrid materials containing three different ZnTPPF_20_CN-to-SnO_2_ ratios (1:4, 1:32, 1:64) were fabricated and tested for acetone detection at 120 °C, both under dark conditions and LED illumination. The sensing behavior of these hybrids was compared with that of previously studied SnO_2_ composites, incorporating physisorbed, unsubstituted ZnTPPF_20_. Among the tested ratios, the 1:32 ZnTPPF_20_CN/SnO_2_ demonstrated superior acetone sensitivity compared to its unmodified counterpart, despite showing a lower intrinsic conductivity in air and a reduced electron transfer efficiency. Density functional theory (DFT) calculations provided insights into the possible anchoring modes and interfacial electronic interactions, helping to rationalize this counterintuitive observation. The enhanced sensing response was attributed to a more favorable balance between charge injection and the availability of SnO_2_ electronic states, facilitated by the chemisorbed anchoring of ZnTPPF_20_CN. Overall, our findings highlight the importance of molecular engineering, particularly in terms of molecular design, loading ratio, and anchoring mechanism, in modulating charge dynamics and optimizing the sensing efficiency of porphyrin/SnO_2_ nanocomposites.

## 1. Introduction

Chemoresistors are a class of chemical sensors whose operation relies on the direct chemical interaction between the sensing material and the target analyte, resulting in measurable changes in the material’s electrical resistance [1]. These devices are primarily employed for gas detection and are capable of sensing both oxidizing and reducing species, such as volatile organic compounds (VOCs) and NO_x_. Thanks to their high sensitivity and relatively simple design, chemoresistive sensors find applications in a wide range of fields, including medical diagnostics, environmental monitoring, and industrial process control [2,3,4,5]. Among the most widely used materials, semiconducting metal oxides (MOS) play a pivotal role, with tin dioxide (SnO_2_) serving as a prototypical example due to its excellent sensitivity [1,6]. However, MOS-based sensors exhibit several drawbacks, including cross-sensitivity, humidity interference, and the need for high operating temperatures [7,8,9]. Therefore, to address these challenges, significant efforts have been focused on tailoring the metal oxides, either as single-phase compounds or in combination with other functional materials, to improve their selectivity, sensitivity, and stability [10,11]. In particular, MOS have been explored in combination with graphene oxide (GO) [12], metal–organic frameworks (MOFs) [13,14], and metal nanoparticles [15,16]. A comparative summary of some recent literature data regarding MOS-based sensors is reported in Table S1.

In recent years, the emergence of conductive polymers [17] and molecularly engineered organic materials has opened new avenues for the development of chemoresistive sensors [18]. Compared to inorganic metal oxides, these materials offer two main advantages: they can operate efficiently at room temperature and, more importantly, their chemical sensitivity can be finely tuned by exploiting the vast diversity of synthetically accessible molecular structures. In this context, the integration of porphyrinoid compounds with MOS is an attractive strategy, owing to the catalytic properties of porphyrins that can enhance the sensing response [6]. Porphyrins are macrocyclic organic molecules characterized by a highly conjugated aromatic core containing 18 π-electrons, which provides them with excellent chemical and thermal stability, as well as good solubility [19]. Their structure is also remarkably versatile: the macrocycle features eight β-pyrrolic positions and four *meso* positions available for substitution, and the central cavity can host a wide range of metal ions, which may further coordinate one or two axial ligands [20,21]. Thanks to this adaptability, porphyrins have been developed for a variety of applications, ranging from catalysis [22,23] to optoelectronics [24,25,26], dye sensitized solar cells (DSSCs) [27,28,29], artificial photosynthesis (DSPECs) [30,31], and sensing [6,32]. Indeed, their structural tunability enables extensive chemical modification, offering the possibility to finely tailor the sensor’s selectivity toward specific analytes while simultaneously reducing the operating temperature [33].

Very recently, we investigated nanocomposites of SnO_2_ with 5,10,15,20-tetraphenylporphyrin Zn(II) (ZnTPP) and its perfluorinated analogue, 5,10,15,20-tetrakis-(pentafluorphenyl)porphyrin Zn(II) (ZnTPPF_20_), for the detection of gaseous acetone at 120 °C [34]. The ZnTPFF_20_/SnO_2_ 1:32 nanocomposite showed an outstanding sensing performance compared to pristine SnO_2_. Experimental characterizations and density functional theory (DFT) calculations revealed that the porphyrin–SnO_2_ interaction was primarily physical in nature, and that perfluorination provided an optimal balance between porphyrin electron injection and available MOS states, which is crucial to increase the conductivity of the material. Notably, we also observed that upon LED illumination, the sensing of ZnTPFF_20_/SnO_2_ 1:32 worsened due to the detrimental effect of the overfilling of SnO_2_ states coupled with enhanced electron scattering.

**Figure 1:**
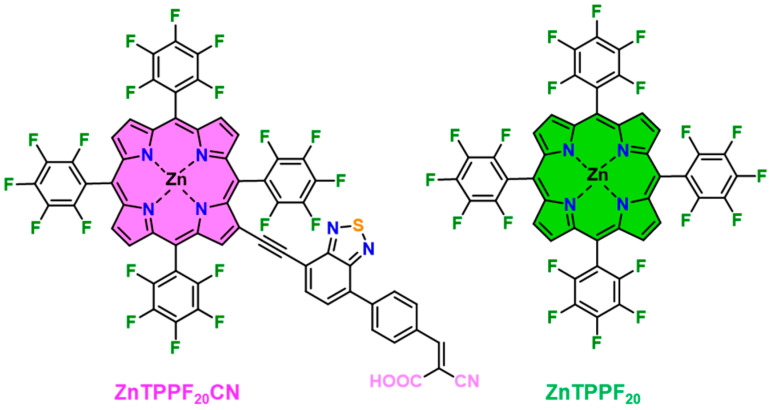
Investigated Zn(II) porphyrins.

Building upon this rationale, the present work explores the integration of perfluorinated Zn(II) porphyrins with SnO_2_ for low-temperature acetone sensing, focusing on hybrids incorporating ZnTPPF_20_CN (Figure 1).

We modified ZnTPPF_20_ by introducing in the β-pyrrolic position of the core an ethynylphenyl-cyanoacrylic pendant as an acceptor and anchoring group. Then we added a benzothiadiazole (BTD) spacer, which is known for its strong electron-withdrawing properties and for its ability to increase light harvesting. Moreover, it enhances charge separation, thus improving the directionality of charge injection in n-type semiconductors from the excited state [27]. In contrast to the physisorption observed for pristine ZnTPPF_20_ on SnO_2_, the introduction of this anchoring group enables chemisorption [31], which is expected to influence the sensing behaviour of the resulting nanocomposites.

Here, we prepared hybrid materials with three different ZnTPPF_20_CN/SnO_2_ weight ratios (1:4, 1:32, and 1:64), deposited them onto glass interdigitated electrodes (IDEs), and measured their acetone sensing performance at 120 °C, both in the dark and under LED illumination, to enable a meaningful comparison with previously reported results for ZnTPPF_20_. Then, we carried out DFT calculations to investigate the interaction between the cyanoacrylic-functionalized porphyrins and the SnO_2_ surface, considering different possible anchoring modes and evaluating their influence on the electronic properties and sensing behavior of the hybrid materials.

## 2. Results

### 2.1. Acetone Sensing

**Figure 2:**
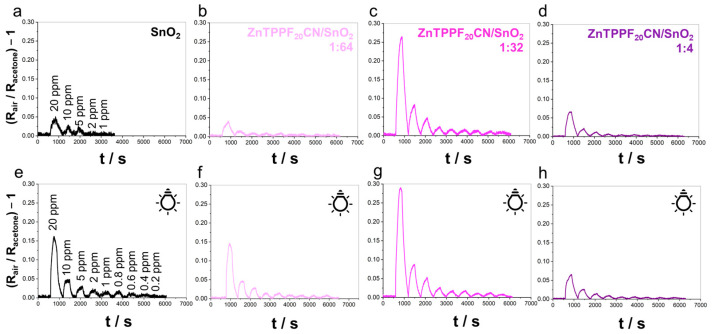
Comparison of sensing responses toward acetone of pristine (**a**,**e**) SnO_2_ and (**b**–**d**,**f**–**h**) ZnTPPF_20_CN/SnO_2_-based composites in simulated air (80% N_2_–20% O_2_), both in dark and upon LED light illumination. Operating temperature = 120 °C, RH% < 2%.

**Figure 3:**
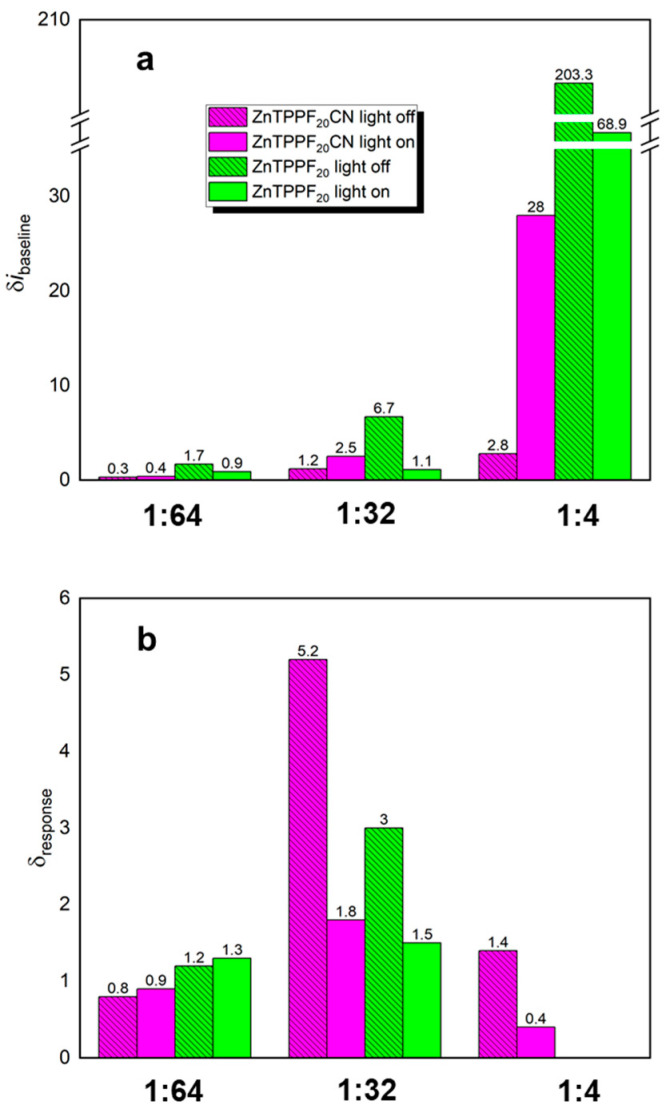
Comparison in dark or under LED irradiation of the investigated ZnTPPF_20_CN/SnO_2_ and the corresponding ZnTPPF_20_/SnO_2_ ones: (**a**) i_baseline_ increment vs. pure SnO_2_ (δi_baseline_), and (**b**) response intensity at 20 ppm increment vs. pure SnO_2_ (δ_response_).

The sensing properties of the ZnTPPF_20_CN/SnO_2_ nanocomposites toward gaseous acetone were evaluated under both dark and LED illumination conditions in simulated air (80% N_2_–20% O_2_). Measurements were carried out by exposing the sensors to acetone concentrations ranging from 20 ppm to 200 ppb at an operating temperature (OT) of 120 °C and under a relative humidity (RH%) below 2%. Figure 2 shows the sensing performances of ZnTPPF_20_CN/SnO_2_ hybrids in the dark and under LED illumination, whereas Figure 3 and Table S2 compare the relative increment of baseline current (in the absence of acetone, i_baseline_) and response intensity at 20 ppm acetone for both ZnTPPF_20_/SnO_2_ and ZnTPPF_20_CN/SnO_2_ hybrids with respect to the pristine SnO_2_ without the porphyrin addition. Moreover, the corresponding calibration curves are reported in Figure S1.

Before testing the electrode response to acetone, the baseline currents (i_baseline_) were measured in simulated air. As previously observed for ZnTPPF_20_/SnO_2_ composites [34], the presence of ZnTPPF_20_CN porphyrin increases i_baseline_ relative to bare SnO_2_ under dark conditions, with the increment (di_baseline_) becoming more pronounced as the porphyrin loading increases (0.3 at 1:64 → 1.2 at 1:32 → 2.8 at 1:4, magenta striped bars in Figure 3a). Under LED illumination, this enhancement is significantly amplified (0.4 at 1:64 → 2.5 at 1:32 → 28.0 at 1:4, solid magenta bars in Figure 3a). These experimental outcomes suggest that, in the absence of acetone, effective electron transfer occurs from the porphyrin to SnO_2_, leading to increased conductivity. Moreover, LED excitation boosts this process: the improved photoresponse is attributed to the BTD moiety in ZnTPPF_20_CN, which broadens the absorption spectrum and provides better overlap with the LED emission at 455 nm, as confirmed by the UV-Vis spectrum (Figure S2). Furthermore, for the ZnTPPF_20_/SnO_2_ composite (depicted by the green striped bars in Figure 3a and Table S2), a progressive increase in di_baseline_ values is evident, rising from 1.7 at 1:64 to 6.7 at 1:32 and becoming markedly pronounced at the 1:4 ratio (203.3). This enhancement is ascribed to the high electron-donating propensity of the perfluorinated porphyrin, which facilitates efficient electron transfer to the SnO_2_ matrix [34]. A comparable trend was also observed under LED light; however, the overall increment exhibited a diminished magnitude relative to that recorded under dark conditions due to limited spectral overlap (Figure S2) between the absorption profile of the ZnTPPF_20_/SnO_2_ system and the emission spectrum of the employed LED source, which consequently reduces the efficiency of photoexcitation.

Upon exposure to acetone, composites with lower porphyrin content (1:64) display negligible sensing responses under both conditions compared to bare SnO_2_ (Figure 2). Actually, an increment value of around 1 is observed (Figure 3b). Notably, the 1:32 ZnTPPF_20_CN/SnO_2_ composite shows a clear sensing response increment under both dark and LED illumination (Figure 2 and Figure 3b, striped and solid magenta bars, respectively), confirming this ratio as optimal, consistent with previous findings for graphene oxide/SnO_2_ [35] and ZnTPPF_20_/SnO_2_ composites [34]. Indeed, Figure 3b clearly demonstrates that 1:32 ZnTPPF_20_CN/SnO_2_ consistently outperforms ZnTPPF_20_/SnO_2_ in acetone sensing both in the dark and under LED light. Moreover, in the case of the highest porphyrin loading (1:4), ZnTPPF_20_CN exhibits a measurable sensing response, while the ZnTPPF_20_/SnO_2_ hybrids show no detectable activity, in agreement with their high baseline current values (Figure 3a and Table S2). Overall, elevated i_baseline_ values systematically lead to reduced sensing performance across all weight ratios [34].

### 2.2. Physicochemical Characterization of the 1:32 ZnTPPF_20_CN/SnO_2_ Composite

**Figure 4:**
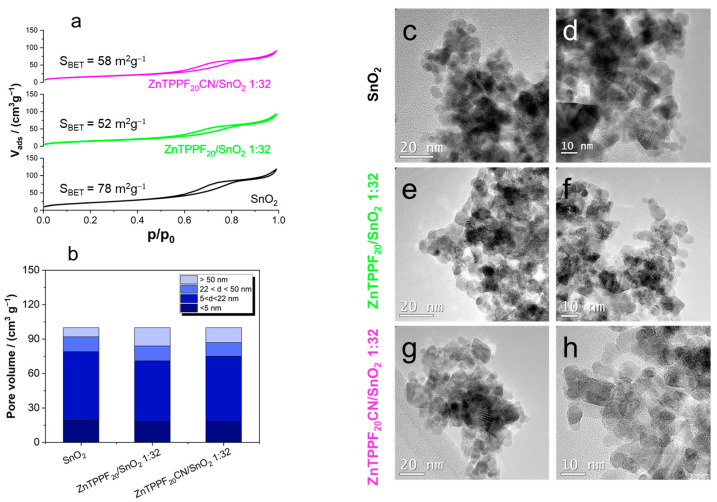
(**a**) BET isotherms and (**b**) pore distribution by BJH analysis. HRTEM images of (**c**,**d**) SnO_2_, (**e**,**f**) ZnTPPF_20_/SnO_2_, and (**g**,**h**) ZnTPPF_20_CN/SnO_2_ composites.

We conducted a comprehensive characterization of the best-performing ZnTPPF_20_CN/SnO_2_ composite to have a meaningful comparison with pristine SnO_2_ and the ZnTPPF_20_/SnO_2_ hybrid. Figure 4a displays the nitrogen adsorption–desorption isotherms of pristine SnO_2_, ZnTPPF_20_/SnO_2_ 1:32, and ZnTPPF_20_-CN/SnO_2_ 1:32 composites, respectively.

The curves reveal typical type IV isotherms with H3 hysteresis loops, indicating the presence of mesoporous structures. The specific surface areas (S_BET_) are 78 m^2^ g^−1^ for SnO_2_, 52 m^2^ g^−1^ for ZnTPPF_20_/SnO_2_, and 58 m^2^ g^−1^ for ZnTPPF_20_CN/SnO_2_, showing a slight decrease upon incorporation of the macrocyclic components, as expected.

Concerning the pore volume distribution (Figure 4b), both 1:32 ZnTPPF_20_CN/SnO_2_ and ZnTPPF_20_/SnO_2_ composites retain significant mesoporosity, with contributions from pores in the 2–50 nm range dominating the total porosity.

Moreover, HRTEM images (Figure 4c–f) confirm that both composites preserve the nanoparticle morphology of the pristine oxide, with no evident aggregation after functionalization. The perfluorinated Zn(II) porphyrins appear to be homogeneously distributed over the oxide nanoparticles, contributing to the maintenance of porous structures. This evidence is fully corroborated by EDS analyses (see Figure S3), showing a homogeneous distribution of both Zn and F species at all porphyrin loadings, consistent with the expected F/Sn and Zn/Sn ratios (Table S3).

**Figure 5:**
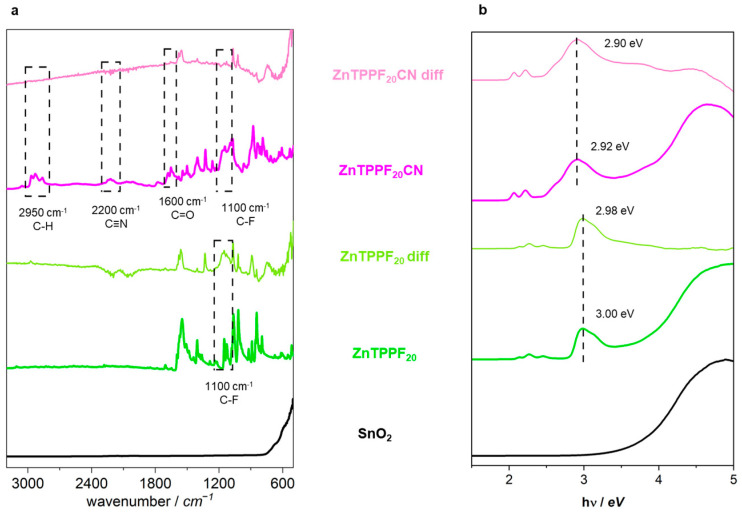
Comparison of (**a**) FTIR and (**b**) DRS spectra of pristine materials and the corresponding ones obtained as a difference between the composites and bare SnO_2_.

The optical characterizations were carried out by attenuated total reflectance–Fourier transform infrared (ATR-FTIR) spectroscopy and Diffuse reflectance spectroscopy (DRS). Subtraction of the spectrum of pure SnO_2_ from that of the hybrid allowed us to evidence the contribution of the porphyrins alone (“ZnTPPF_20_CN diff” and “ZnTPPF_20_ diff” patterns in Figure 5a). The comparison of the differential spectra with those of pristine porphyrins helped us to gain insight into porphyrin–SnO_2_ interaction.

The spectrum of ZnTPPF_20_CN exhibits three peculiar bands at about 2950, 2200, and 1600 cm^−1^, due to C-H, C≡N, and C=O stretching (dashed pink rectangles in Figure 5a) [36]. Moreover, as for ZnTPPF_20_, C-F stretching modes at 1000–1100 cm^−1^ are visible (dashed pink and green rectangles in Figure 5a). All these signals disappear in the difference spectrum, thus confirming the effective interaction between ZnTPPF_20_CN and SnO_2_.

As for the DRS spectra (Figure 5b), they retain the typical pattern observed in solution (Figure S2), with an intense Soret band at about 3.0 eV and two Q bands at lower energy. Moreover, the bands for pure ZnTPPF_20_CN are broader and red-shifted compared to those of ZnTPPF_20_ also in the solid state, in agreement with the extended π-conjugation provided by the presence of the BTD-equipped anchoring group [37]. Finally, the difference spectra show a slight broadening and a shift at higher energy of the Soret band, further supporting the formation of the hybrid [38].

### 2.3. Ab Initio Calculations

The ab initio investigation began with the study of bulk SnO_2_ and its (110) surface, which is reported in the literature as the most stable facet [39]. The spectral properties of the surface were calculated using a symmetric slab composed of 15 layers, in which only the four most external layers were allowed to relax. The results show the appearance of discrete states within the band gap, which, as revealed by the projected band structure analysis, belong to unsaturated Sn and O atoms on the surface (Figure S4).

**Figure 6:**
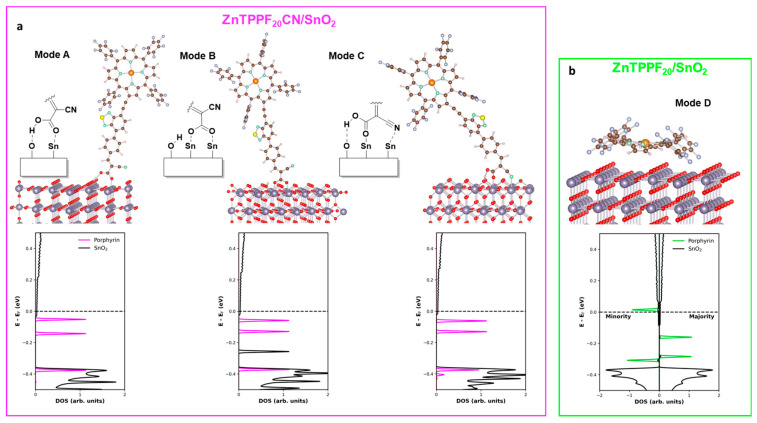
Possible adsorption modes of (**a**) ZnTPPF_20_CN and (**b**) ZnTPPF_20_ on SnO_2_ surface, and corresponding projected DOS.

ZnTPPF_20_CN can exist in at least two configurational isomers, Z and E, arising from the rigid rotation around the C=C double bond of the side chain (Figure S5, Charts S1 and S2). Both isomers were optimized with the SIESTA code, revealing that the E-isomer is more stable with an energy difference of 0.26 eV. According to a Boltzmann distribution, the probability of the E-isomer being present under thermodynamic control at room temperature exceeds 99%. This result is further supported by literature findings, according to which the E-isomer is the predominant product in the Knoevenagel condensation [40]. However, to account for potential reconfiguration mechanisms that may occur upon interaction with the surface, the adsorption of both isomers on the SnO_2_ surface was examined. To assess how the presence of the pendant group may affect the adsorption pathway and, thus, potentially influence the overall performance of the system, three different adsorption modes of the ZnTPPF_20_CN were hypothesized (Figure 6a).

In order for the E-isomer to adsorb onto the surface without involving the porphyrinic moiety, it is reasonable to assume that the chemisorption takes place via the carboxylic group. Following a previous report on the adsorption of formic acid on the SnO_2_ (110) surface [41], we also considered for ZnTPPF_20_CN two main adsorption manners: the monodentate mode (A) and the bidentate mode (B). In the monodentate configuration, the carbonyl oxygen interacts with a positively charged surface Sn atom, while the acidic hydrogen forms a bridge with a surface oxygen atom bearing a partial negative charge. In the bidentate mode, instead, the carboxylic (COOH) group is deprotonated and binds to two surface Sn atoms, while the proton is adsorbed on a nearby surface oxygen. For these two adsorption configurations, the calculated adsorption energies are −1.61 eV for mode A and −2.01 eV for mode B, respectively (Table 1).

The Z-isomer can also approach the surface through the cyano group, whose anchoring ability in cyanoacrylic-type systems has already been established on anatase TiO_2_ (101) surface [38]. After structural optimization, we found that the pendant moiety chemisorbs in a bidentate fashion, involving both the carboxylic and the cyano groups. Additionally, the acidic hydrogen coming from the carboxyl is bridged with a surface Sn atom. For this configuration, referred to as Mode C, the calculated adsorption energy is −1.87 eV (Table 1).

The interaction between the ZnTPPF_20_ molecule and the SnO_2_ surface has already been analyzed in our earlier work [32]. In that study, a physisorption configuration was proposed in which the porphyrin macrocycle lies flat on the SnO_2_ surface (Figure 4b). In this orientation, the conjugated π-system of the porphyrin is expected to interact with the charged atoms on the substrate surface. However, for the sake of consistency, in the present work, the system was re-simulated, increasing the thickness of the substrate. The calculated adsorption energy for this system is −0.15 eV (Table 1), indicating a stable adsorption geometry, although markedly less stable than the upright configurations A–C. Compared with our previous work—where we examined the same flat-lying geometry for different molecules—we note that we now include a correction for the basis-set superposition error. This error affects any atomic-orbital basis calculation using moderately sized basis sets, such as that required here by the large size of the simulated system. It differs substantially between flat-lying and upright orientations but remains consistent within each class. After applying the correction according to the counterpoise scheme [39], we obtain a binding energy consistent with a physisorption regime (0.1–0.8 eV), as expected for a π–stacking interaction with limited charge transfer to the surface. The latter is found to be comparatively larger than in the upright configurations (~0.5 e^−^) but distributed over a much broader contact area.

We employed projected-DOS to gain insights into the nature of the interaction between ZnTPPF_20_CN and the SnO_2_ substrate. In all investigated adsorption configurations, a charge transfer from the porphyrin to the substrate was observed, resulting in a partial filling of the SnO_2_ conduction band. We found that when the porphyrin is chemisorbed via the pendant group, the amount of transferred charge increases from 0.20 e^−^ in mode B, to 0.24 e^−^ in mode C, while mode A shows the highest value of 0.38 e^−^ (Table 1).

On the other hand, the projected-DOS analysis on ZnTPPF_20_/SnO_2_ revealed that the system is spin polarized. As shown in the spin-resolved DOS (Figure 4b), the originally highest occupied states of the porphyrin lose degeneracy. One spin channel shifts above the Fermi level, becoming partially depopulated. The majority spin component peak remains filled, being located below the Fermi level.

These findings support the higher charge injection capability of the ZnTPPF_20_, estimated to be 0.51 e^−^, in comparison to the values obtained for the ZnTPPF_20_CN configurations.

## 3. Discussion

At the lowest porphyrin loading (1:64), ZnTPPF_20_/SnO_2_ hybrids show higher sensing performance due to efficient electron injection into SnO_2_, unlike the weaker response observed for ZnTPPF_20_CN. However, due to the small amount of porphyrin, the overall electron transfer to SnO_2_ remains minimal, resulting in a response comparable to that of bare SnO_2_ (d_response_ = 0.8–1.3, Figure 3b).

The behavior of the 1:32 hybrid, however, is more intriguing. Based on the increase in baseline current under dark conditions, chemisorbed ZnTPPF_20_CN appears to transfer electrons to SnO_2_ less efficiently than physisorbed ZnTPPF_20_ (di_baseline_ 1.2 vs. 6.7, Figure 3a), as supported by Bader analysis, which shows higher transferred charge and baseline current for ZnTPPF_20_. Therefore, one might expect ZnTPPF_20_/SnO_2_ to display superior sensing performance, yet the opposite occurs: ZnTPPF_20_CN/SnO_2_ exhibits a stronger response (d_response_ 5.2 vs. 3, Figure 3b). This outcome can be explained by the fact that, as demonstrated in our recent work [34], the sensing behavior depends on achieving an optimal balance between charge injection and the availability of SnO_2_ electronic states. Thus, the chemical anchoring mode of the ZnTPPF_20_CN enables sufficient electron transfer while maintaining this balance.

Finally, the 1:4 ratio for ZnTPPF_20_CN proves a moderate increment (d_response_ = 1.4) to sensing with respect to pristine SnO_2_, as the excessive porphyrin content hampers acetone detection by SnO_2_ with respect to that of the 1:32 ratio. This result can be attributed to a “shielding” effect caused by the steric hindrance of ZnTPPF_20_CN, which makes the SnO_2_ surface less prone to react with the reducing agent. Specifically, the porphyrin molecules may adopt a tilted orientation rather than lying flat on the surface (as in the case of ZnTPPF_20_, see Figure 6), as their concentration increases, leading to higher surface coverage and p-p aggregation, also confirmed by the broad bands in DRS analysis [38]. This molecular arrangement creates a surface blocking effect [29], which limits electron accessibility to the SnO_2_ surface and, thereby, hinders the sensing process. In contrast, ZnTPPF_20_ exhibits no sensing response, consistent with the excessively high baseline current values.

Under LED light, only ZnTPPF_20_CN/SnO_2_ hybrids show an increased baseline (Table S2, 6th column), due to the matching between the light source emission at 455 nm and the UV-Vis absorption of the porphyrin (Figure S2). On the contrary, LED illumination is detrimental for ZnTPPF_20_/SnO_2_ nanocomposites (Figure 3a). However, in the presence of acetone (Figure 3b), both ZnTPPF_20_/SnO_2_ and ZnTPPF_20_CN/SnO_2_ hybrids show similar (for 1:64 ratio) or reduced (1:32 and 1:4 ratios) performances passing from dark to LED illumination. This experimental outcome corroborates the intimate relationship between baseline current and sensing response, notwithstanding the broader absorption of the cyanoacrylic-functionalized porphyrin.

## 4. Materials and Methods

### 4.1. Synthesis of ZnTPPF_20_CN, SnO_2_ and Hybrid Materials

ZnTPPF_20_CN was synthesized by modifying and optimizing a reported preparation [37]. The optimization focused on the final step of the synthesis, specifically the Knoevenagel condensation. In this step, the solvent was changed from a mixture of CH_3_CN/CHCl_3_ to toluene, leading to an increase in yield from 49% to 73%. The experimental details are in Supplementary Materials (Scheme S1).

SnO_2_ nanoparticles were prepared as reported elsewhere [12,35], and 1:64, 1:32, and 1:4 ZnTPPF_20_CN/SnO_2_ nanocomposites were assembled using the straightforward dissolution/deposition approach already used for ZnTPPF_20_/SnO_2_ hybrids [34].

### 4.2. Characterization of ZnTPPF_20_CN and of the 1:32 ZnTPPF_20_CN/SnO_2_ Composite

ZnTPPF_20_CN and its precursors were characterized by ^1^H- and ^19^F-NMR spectroscopy (Figures S6–S16). NMR spectra were recorded on a Bruker Avance DRX-400 spectrometer (Billerica, MA, US) using CDCl_3_ and THF-d_8_ as solvents (Sigma-Aldrich, St. Louis, MI, USA). The UV-Vis absorption spectrum of ZnTPPF_20_CN (Figure S2) was acquired at room temperature in THF with a Shimadzu UV-3600 spectrophotometer (Tokyo, Japan), employing quartz cuvettes with an optical path length of 1 cm. ESI-ITMS spectrum of ZnTPPF_20_CN (Figure S17) was acquired on a Bruker Daltonics ICR-FTMS APEXII with an electrospray ionization source.

The most efficient 1:32 ZnTPPF_20_CN/SnO_2_ nanocomposite was comprehensively characterized using a range of experimental techniques. High-resolution transmission electron microscopy (HRTEM) was carried out on an FEI TECNAI G2 F20 instrument (Thermo Fisher Scientific, Waltham, MA, USA) operating at 200 kV and equipped with an S-Twin lens, providing a point resolution of 0.24 nm. TEM grids were prepared by depositing a suspension of nanoparticles in ethanol onto holey carbon–supported copper grids, followed by drying at room temperature in air.

SEM-EDX analyses were performed with a Hitachi TM-4000 (Tokyo, Japan) scanning electron microscope equipped with a 4-quadrant BSE (backscattered electrons) detector, a low-vacuum SE (secondary electrons) detector, and an Oxford AztecOne EDX (Abingdon-on-Thames, UK). Map acquisitions were performed at an acceleration voltage of 15 kV and at 300× magnification.

The specific surface area and porosity were determined using the multipoint Brunauer–Emmett–Teller (BET) method with N_2_ adsorption–desorption isotherms at 77 K on a Tristar II 3020 (Micromeritics) apparatus (Norcross, GA, USA). Total pore volume was estimated from desorption isotherms using the Barrett–Joyner–Halenda (BJH) method. Prior to measurements, the samples were thermally treated at 80 °C overnight to prevent material degradation.

Attenuated total reflectance–Fourier transform infrared (ATR–FTIR) spectra were collected in the 4000–400 cm^−1^ range using a PerkinElmer Frontier spectrometer equipped with a diamond/ZnSe ATR crystal (Waltham, MA, USA). Diffuse reflectance spectra (DRS) were recorded over the 220–2600 nm range with a Shimadzu UV-3600 Plus double-beam UV-Vis–NIR spectrophotometer (Tokyo, Japan) fitted with an integrating sphere (BIS-603). Finely ground powders were pressed into uniform circular pellets (0.2 cm diameter), placed in a quartz cuvette, and positioned against the integrating sphere window for reflectance acquisition. Barium sulfate (BaSO_4_) served as the reflectance standard.

### 4.3. Preparation of the Electrodes

Nanocomposite films were deposited using a hot-spray technique onto glass interdigitated platinum electrodes (Pt-IDEs, purchased from Metrohm, Herisau, Switzerland, Figure S18). Gas sensing experiments were conducted in a previously described custom-made stainless-steel cell (Figure S18) [42,43]. The electrical resistance of the films was monitored under a simulated air atmosphere (80% N_2_–20% O_2_, total flow rate 0.5 L·min^−1^) containing different concentrations of acetone vapor. The acetone flow was adjusted by diluting a 500 ppm stock mixture in N_2_ using Bronkhorst mass flow controllers, while maintaining the overall flow rate constant.

The dynamic sensing response (Figure S18) was measured with an Autolab PGStat30 potentiostat/galvanostat (Eco Chemie, Utrecht, The Netherlands) operated by NOVA 2.0 software, under a constant bias of +1.0 V. Sensor output was expressed as (R_air_/R_acetone_) − 1, where R_air_ and R_acetone_ are the film resistances in synthetic air and acetone, respectively.

All measurements were carried out at (120 ± 2) °C and <2% relative humidity (verified using a hygrometer placed at the outlet of the sensing chamber). For selected experiments, additional irradiation was applied using either a blue LED (THORLABS, λ = 455 nm, 2 W) or a UV lamp (Jelosil HG500 iron halide mercury arc lamp, 500 W, emission range 350–450 nm, incident power density 30 mW·cm^−2^). All tests are conducted in triplicate, and the error bars are at most 5% of the sensor response.

Since the SnO_2_ batch used to prepare the ZnTPPF_20_CN/SnO_2_ hybrids in this study differs from the one previously employed for the ZnTPPF_20_/SnO_2_ hybrids [34], we normalized both the baseline currents and sensor responses by dividing them by the corresponding values of the respective SnO_2_ batch. The resulting normalized data are presented as the increment of baseline current relative to SnO_2_ (di_baseline_) and as the increment of response intensity at 20 ppm relative to SnO_2_ (d_response_), as reported in Table S2 and Figure 6.

### 4.4. Computational Details

The theoretical study of the ZnTPPF_20_-CN and its adsorption onto the SnO_2_ surface was carried out using density functional theory (DFT) as implemented in the SIESTA code (v5.2.2) [44]. Calculations used the Perdew–Burke–Ernzerhof (PBE) exchange-correlation functional and a double-zeta polarized (DZP) basis set with default parameters. Norm-conserving pseudopotentials were generated using the Troullier–Martins formalism, and real-space integrations were performed with a grid equivalent to a plane-wave cutoff of 450 Ry. The SnO_2_ bulk structure was modeled using a tetragonal rutile-like unit cell (2 Sn and 4 O atoms) with optimized lattice constants (a = b = 4.86 Å, c = 3.29 Å), and the Brillouin zone was sampled with a 10 × 10 × 14 Monkhorst–Pack grid. Surface models focused on the (110) facet, represented by a 6-layer slab in which only the top four layers were relaxed. A 30 Å vacuum region was introduced to prevent spurious periodic interactions. The surface area was selected to accommodate the entire porphyrin molecule. For ZnTPPF_20_CN adsorption, the slab model included 576 atoms (192 SnO_2_ units), while the system with ZnTPPF_20_ contained 648 atoms (216 SnO_2_ units). The electron density distribution of the system was analyzed within the framework of the Quantum Theory of Atoms in Molecules (QTAIM) [45] using the Critic2 code [46], and the total energies used in computing the adsorption energies are corrected for the basis set superposition error (BSSE).

## 5. Conclusions

The combined experimental and theoretical investigation demonstrates that functionalization of SnO_2_ with perfluorinated Zn(II) porphyrins bearing different substituents can significantly enhance acetone sensing performances, particularly at an optimal loading ratio. The incorporation of the cyanoacrylic anchoring group promotes effective chemisorption on the SnO_2_ surface, ensuring balanced charge transfer while preserving a limited yet sufficient availability of both the semiconductor’s electronic states and its catalytic sites, which are essential for gas detection. Optical and structural characterizations confirm strong porphyrin–oxide coupling without compromising the mesoporous architecture, whereas DFT calculations reveal that the pendant group ensures stable adsorption and efficient electron injection at the interface. Furthermore, LED illumination markedly improves the photoresponse when its emission spectrum overlaps with the porphyrin absorption band. Overall, these findings demonstrate that rational molecular design of porphyrinic sensitizers represents an effective strategy to control charge transfer processes and optimize light-assisted gas sensing in metal oxide–porphyrin hybrid materials.

## Figures and Tables

**Table 1:** Adsorption energy and transferred charge for the different adsorption modes on the SnO_2_ surface of ZnTPPF_20_CN and ZnTPPF_20_.

Molecule	Mode	Adsorption Energy eV	Integrated Charge e^−^
ZnTPPF_20_CN	A	−1.61	0.38
	B	−2.01	0.20
	C	−1.87	0.24
ZnTPPF_20_	D	−0.15	0.51

## Data Availability

The data that support the findings of this study are available from the corresponding author upon reasonable request.

## Supplementary Materials

The following supporting information can be downloaded at: https://www.mdpi.com/article/10.3390/molecules30244749/s1. Table S1: A comparative summary of some recent literature data regarding MOS-based sensors; Table S2: Baseline current values (i_baseline_), i_baseline_ increment vs. pure SnO_2_ (di_baseline_), response intensity at 20 ppm, and response intensity at 20 ppm increment vs. pure SnO_2_ (d_response_), for ZnTPPF_20_CN/SnO_2_ and ZnTPPF_20_/SnO_2_ nanocomposites in dark and under LED light irradiation. Data in green from ref. [34] of the main paper; Table S3: Theoretical vs. experimental (by EDS) Zn/Sn and F/Zn atomic ratios for ZnTPPF_20_CN/SnO_2_ hybrids at all porphyrin loadings; Figure S1: Calibration curves for ZnTPPF_20_CN/SnO_2_ hybrids; Figure S2: Normalized UV-Vis spectra of ZnTPPF_20_ and ZnTPPF_20_CN in THF; Figure S3: EDS analyses for ZnTPPF_20_CN/SnO_2_ hybrids; Figure S4: Fatband structure of a 15 layers symmetrical slab of SnO_2_, blue and green represents contribution coming from Sn and O surface atoms, respectively. On the right panel a comparison between the (110) surface DOS (blue) and the bulk DOS (black) of the rutile phase of SnO_2_; Figure S5: Optimized structures and energy of E and Z isomers of ZnTPPF_20_CN; Figure S6: ^1^H-NMR of ZnTPPF_20_ in CDCl_3_; Figure S7: ^19^F-NMR of ZnTPPF_20_ in CDCl_3_; Figure S8: ^1^H-NMR of ZnTPPF_20_Br in CDCl_3_; Figure S9: ^19^F-NMR of ZnTPPF_20_ in CDCl_3_; Figure S10: ^1^H-NMR of ZnTPPF_20_Si(hex)_3_ in CDCl_3_; Figure S11: ^19^F-NMR of ZnTPPF_20_Si(hex)_3_ in CDCl_3_; Figure S12: ^1^H-NMR of ZnTPPF_20_CH in CDCl_3_; Figure S13: ^1^H-NMR of ZnTPPF_20_CHO in CDCl_3_; Figure S14: ^19^F-NMR of ZnTPPF_20_CHO in CDCl_3_; Figure S15: ^1^H-NMR of ZnTPPF_20_CN in D_2_O; Figure S16: ^19^F-NMR of ZnTPPF_20_CN in D_2_O; Figure S17:ESI-ITMS spectrum of ZnTPPF_20_CN; Figure S18: Image of the gas sensor testing setup: (A) homemade stainless steel in situ sensor testing cell; (B) gas manifold; (C) cell temperature controller; (D) Autolab potentiostat. Inset: inside of the in situ sensor testing cell: (E) heating plate for temperature control; (F) needle-electrical connectors; (G) Pt-interdigitated electrode covered by the synthesized sensing materials. Chart S1: Structural coordinates of the E isomer of ZnTPPF_20_CN; Chart S2: Structural coordinates of the Z isomer of ZnTPPF_20_CN; Scheme S1: Synthetic protocol to ZnTPPF_20_CN [11,14,35,47,48,49,50,51].
