# Supplementary material for: Chemisorption vs. Physisorption in Perfluorinated Zn(II) Porphyrin–SnO2 Hybrids for Acetone Chemoresistive Detection
Source: Molecules. 2025 Dec 12;30(24):4749. doi: 10.3390/molecules30244749 (PMC12735734; doi:10.3390/molecules30244749)
Supplement: Supplementary file 1 [file molecules-30-04749-s001.zip › molecules-3971800-supplementary.pdf]

Supplementary information

Chemisorption vs. Physisorption in Perfluorinated Zn(II) Porphyrin–SnO_2 Hybrids for Acetone Chemoresistive Detection

Manuel Minnucci [1], Sara Oregioni [1], Eleonora Pargoletti [1,2], Gabriele Di Carlo [1,2], Francesca Tessore [1,2*], Gian Luca Chiarello [1], Rocco Martinazzo [1], Mario Italo Trioni [3] and Giuseppe Cappelletti [1,2]

[1] Dipartimento di Chimica, Università degli Studi di Milano, via Golgi 19, 20133 Milano, Italy
[2] Consorzio Interuniversitario Nazionale per la Scienza e Tecnologia dei Materiali (INSTM), via Giusti 9, 50121 Firenze, Italy
[3] National Research Council of Italy, Institute of Chemical Sciences and Technologies "Giulio Natta", via Golgi 19, 20133 Milano, Italy

Table S1. A comparative summary of some recent literature data regarding MOS-based sensors

Material	Operating temperature (°C)	Signal response ($R_{air}/R_{acetone}$)-1	LOD (ppb)	Ref.
rGO/SnO_2	300	11.0 (100 ppm)	–	[47]
GO/SnO_2 1:32	350	12.5	5	[35]
SnO_2 hollow spheres	200	15.0 (50 ppm)	5000	[48]
ZnO/SnO_2 thick films	180	13 (10 ppb)	–	[49]
MOF-derived hollow SnO_2-ZnO nanoparticles	240	139 (10 ppm)	820	[14]
Rh-doped SnO_2 nanofibers	200	59.6 (50 ppm)	1000	[50]
Larger lateral area SnO_2	250	262 (5 ppm)	40 ppt	[11]

Table S2. Baseline current values ($i_{baseline}$), $i_{baseline}$ increment *vs.* pure SnO_2 ($\delta i_{baseline}$), response intensity at 20 ppm, and response intensity at 20 ppm increment *vs.* pure SnO_2 ($\delta_{response}$), for $ZnTPPF_{20}CN/SnO_2$ and $ZnTPPF_{20}/SnO_2$ nanocomposites in dark and under LED light irradiation. Data in green from ref [34] of the main paper.

material	Light off				LED			
	$i_{baseline}$ (μA)	$\delta i_{baseline}$	$(R_{air}/R_{acetone}) - 1$ at 20 ppm	$\delta_{response}$	$i_{baseline}$ (μA)	$\delta i_{baseline}$	$(R_{air}/R_{acetone})-1$ at 20 ppm	$\delta_{response}$
SnO_2 for $ZnTPPF_{20}CN$	25	–	0.05	–	40	–	0.16	–
SnO_2 for $ZnTPPF_{20}$	3	–	0.60	–	9	–	0.55	–
**$ZnTPPF_{20}CN/SnO_2$**								
1:64	8	*0.3*	0.04	*0.8*	15	*0.4*	0.14	*0.9*
1:32	30	*1.2*	0.26	*5.2*	100	*2.5*	0.29	*1.8*
1:4	70	*2.8*	0.07	*1.4*	800	*20.0*	0.07	*0.4*
**$ZnTPPF_{20}/SnO_2$**								
1:64	5	*1.7*	0.70	*1.2*	8	*0.9*	0.72	*1.3*
1:32	20	*6.7*	1.80	*3.0*	10	*1.1*	0.80	*1.5*
1:4	610	*203.3*	–	–	620	*60.9*	–	–

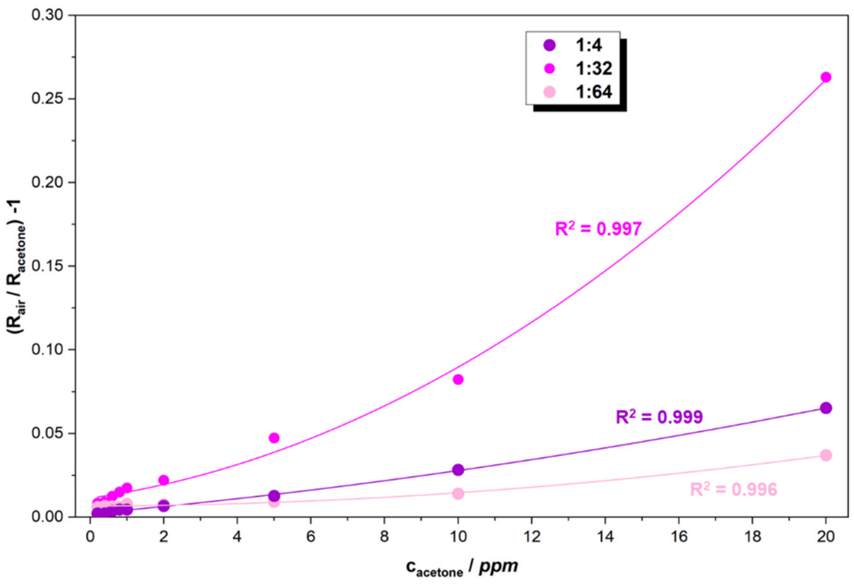

Figure S1. Calibration curves for $ZnTPPF_{20}CN/SnO_2$ hybrids

For all porphyrin-based sensors examined, the slope of the response curve increases non-linearly with analyte concentration. At low gas concentrations, only a limited number of surface adsorption sites are occupied. As the concentration increases, a larger population of adsorbed species alters the surface potential, leading to a reduction in the depletion layer width. Because the surface potential exhibits a non-linear dependence on surface charge [51], the sensor response becomes increasingly pronounced as additional gas molecules are adsorbed, resulting in a progressively steeper slope of the response curve.

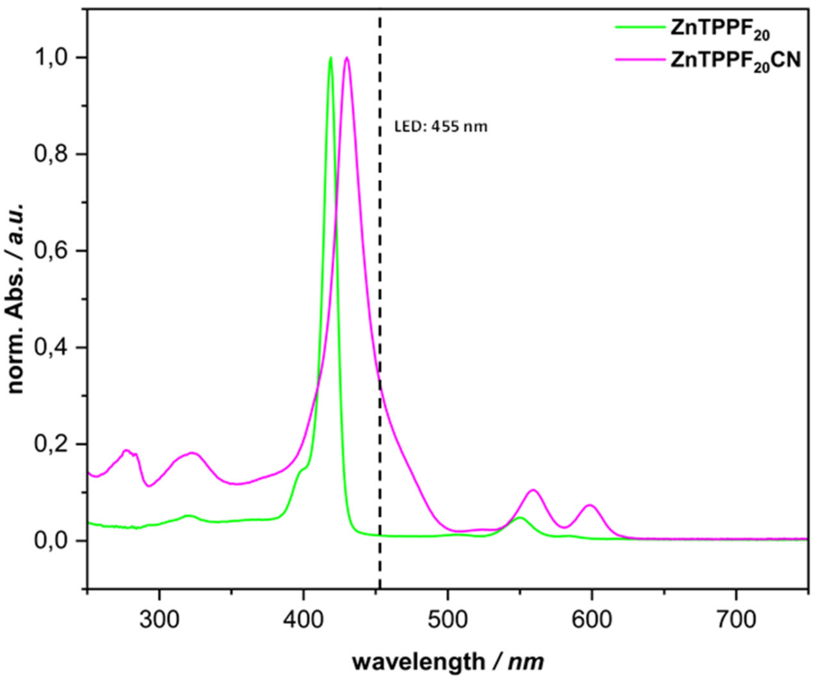

Figure S2. Normalized UV-Vis spectra of $ZnTPPF_{20}$ and $ZnTPPF_{20}CN$ in THF

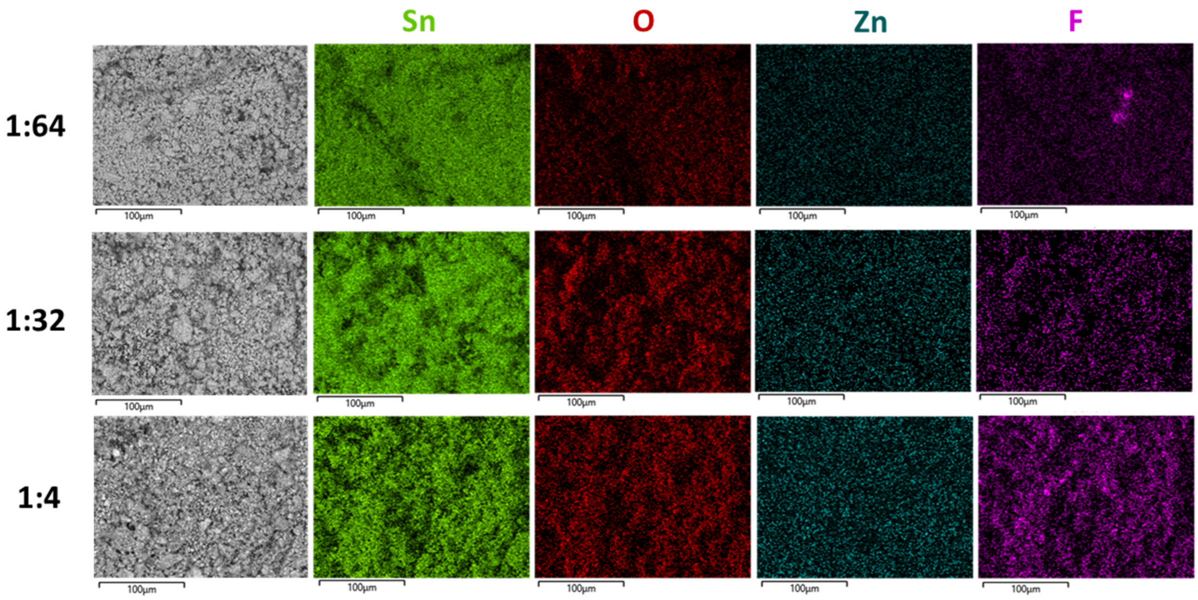

Figure S3. EDS analyses for $ZnTPPF_{20}CN/SnO_2$ hybrids

Table S3. Theoretical vs. experimental (by EDS) Zn/Sn and F/Zn atomic ratios for $ZnTPPF_{20}CN/SnO_2$ hybrids at all porphyrin loadings.

**$ZnTPPF_{20}CN/SnO_2$**	**Zn/Sn**		**F/Sn**	
	Theoretical	Experimental (EDS)	Theoretical	Experimental (EDS)
1:64	0.0017	0.0011	0.034	0.070
1:32	0.0033	0.0022	0.066	0.093
1:4	0.0276	0.0390	0.552	0.470

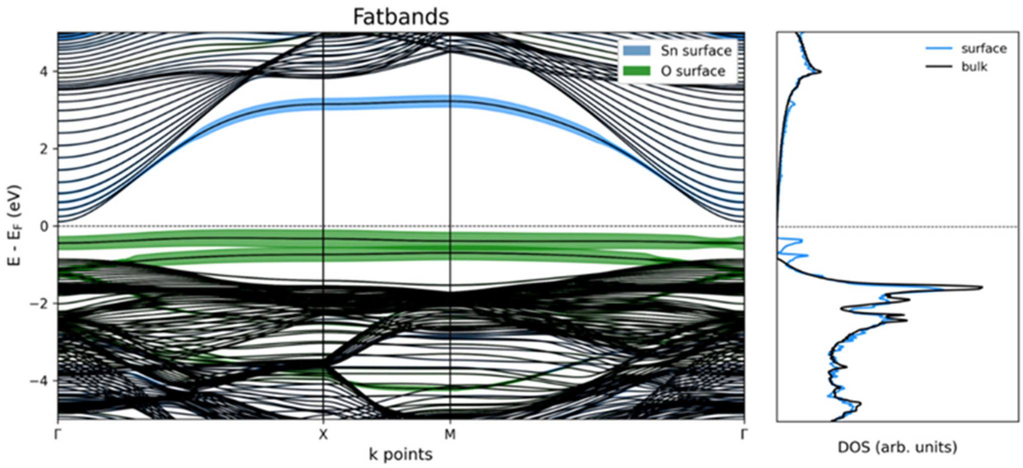

Figure S4. Fatband structure of a 15 layers symmetrical slab of SnO_2, blue and green represents contribution coming from Sn and O surface atoms, respectively. On the right panel a comparison between the (110) surface DOS (blue) and the bulk DOS (black) of the rutile phase of SnO_2.

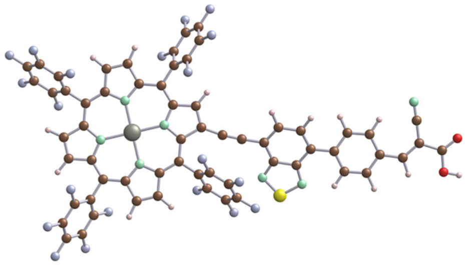

E isomer

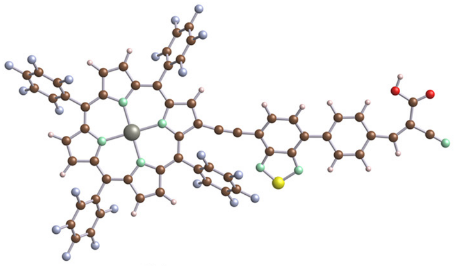

Z isomer

Figure S5. Optimized structures and energy of E and Z isomers of $ZnTPPF_{20}CN$.

$ZnTPPF_{20}CN$_E

Structure parameters

			x	y	z
1	N	N1	-1.28677	0.67261	0.13007
2	N	N2	-3.84658	2.04087	-0.04054
3	N	N3	-5.21795	-0.49937	-0.13880
4	N	N4	-2.66865	-1.86393	-0.02223
5	C	C1	-0.18098	-0.14219	0.15147
6	C	C2	1.04022	0.67334	0.10231
7	C	C3	0.60905	1.99071	0.07842
8	C	C4	-0.82425	1.97184	0.10038
9	C	C5	-1.62598	3.13673	0.07147
10	C	C6	-3.03261	3.15267	0.01019
11	C	C7	-3.83355	4.35987	-0.00265
12	C	C8	-5.13998	3.96460	-0.07870
13	C	C9	-5.13905	2.51537	-0.09233
14	C	C10	-6.30685	1.72584	-0.16002
15	C	C11	-6.32691	0.31595	-0.18411
16	C	C12	-7.53430	-0.48393	-0.23643
17	C	C13	-7.13909	-1.79274	-0.23497
18	C	C14	-5.69122	-1.79239	-0.16114
19	C	C15	-4.89537	-2.95596	-0.10918
20	C	C16	-3.48603	-2.97303	-0.02653
21	C	C17	-2.69449	-4.17657	0.12825
22	C	C18	-1.38919	-3.78069	0.22809
23	C	C19	-1.38307	-2.33544	0.13015
24	C	C20	-0.21426	-1.55047	0.20748
25	H	H1	1.24430	2.87697	0.00750
26	H	H2	-3.44515	5.38033	0.05017
27	H	H3	-6.02900	4.59839	-0.13020
28	H	H4	-8.55463	-0.09223	-0.25812
29	H	H5	-7.77213	-2.68278	-0.28271
30	H	H6	-3.09372	-5.19296	0.18629
31	H	H7	-0.50511	-4.40604	0.38087
32	C	C21	-7.61658	2.43589	-0.19796
33	C	C22	-8.03708	3.25450	0.86786
34	C	C23	-8.48946	2.31959	-1.29670
35	C	C24	-9.26445	3.93117	0.84910
36	C	C25	-9.72306	2.98443	-1.34182
37	C	C26	-10.11050	3.79456	-0.26273
38	C	C27	-0.91815	4.44595	0.10775
39	C	C28	-1.02597	5.37765	-0.94273
40	C	C29	-0.09942	4.80255	1.19743
41	C	C30	-0.35499	6.60817	-0.91797
42	C	C31	0.58642	6.02396	1.24530
43	C	C32	0.45691	6.92970	0.18095
44	C	C33	1.07336	-2.27080	0.40508
45	C	C34	1.63055	-3.08359	-0.59925
46	C	C35	1.78037	-2.14686	1.61398
47	C	C36	2.85338	-3.73682	-0.41432
48	C	C37	3.00856	-2.78872	1.82275
49	C	C38	3.54503	-3.57878	0.79635
50	C	C39	-5.59734	-4.26932	-0.10726
51	C	C40	-5.38656	-5.21919	-1.12519
52	C	C41	-6.49792	-4.61857	0.91758
53	C	C42	-6.04040	-6.45922	-1.13217
54	C	C43	-7.16727	-5.84966	0.93341
55	C	C44	-6.93504	-6.77380	-0.09718
56	Zn	Zn1	-3.25831	0.08524	-0.03067
57	F	F1	-1.79471	5.09449	-2.00918
58	F	F2	-0.47725	7.47115	-1.93400
59	F	F3	1.10520	8.09786	0.21477
60	F	F4	1.35450	6.33324	2.29698
61	F	F5	0.04169	3.95331	2.22975
62	F	F6	-8.14461	1.54650	-2.34177
63	F	F7	-10.52689	2.85727	-2.40426
64	F	F8	-11.28261	4.43455	-0.29368
65	F	F9	-7.24343	3.40625	1.94310
66	F	F10	-9.63486	4.69811	1.88154
67	F	F11	-4.53168	-4.94376	-2.12557
68	F	F12	-5.82277	-7.33891	-2.11755
69	F	F13	-7.56438	-7.95273	-0.09217
70	F	F14	-8.01625	-6.15028	1.92359
71	F	F15	-6.73628	-3.75295	1.91874
72	F	F16	1.28563	-1.37077	2.59218
73	F	F17	3.67129	-2.64343	2.97709
74	F	F18	4.74067	-4.16577	0.95008
75	F	F19	3.40041	-4.47900	-1.39166
76	F	F20	0.98467	-3.23067	-1.76815
77	C	C45	2.39405	0.28885	0.02225
78	C	C46	3.61186	0.11131	-0.06684
79	C	C47	5.01875	-0.00881	-0.09815
80	C	C48	5.85478	0.96680	0.46487
81	C	C49	5.68324	-1.14241	-0.68017
82	C	C50	7.26120	0.85187	0.45900
83	H	H8	5.38884	1.84088	0.93845
84	C	C51	7.13096	-1.26988	-0.65678
85	C	C52	7.95771	-0.23060	-0.09817
86	H	H9	7.83298	1.64979	0.95131
87	N	N5	5.05636	-2.17507	-1.27494
88	N	N6	7.55777	-2.42536	-1.21826
89	S	S1	6.22502	-3.22136	-1.70918
90	C	C53	9.42904	-0.27390	-0.10705
91	C	C54	10.18009	0.92599	-0.00485
92	C	C55	10.14761	-1.48975	-0.22203
93	C	C56	11.56941	0.92408	0.01587
94	H	H10	9.65896	1.89179	0.02594
95	C	C57	11.53950	-1.49406	-0.20317
96	H	H11	9.59078	-2.42787	-0.33958
97	C	C58	12.28983	-0.29649	-0.07623
98	H	H12	12.10719	1.87775	0.09351
99	H	H13	12.07623	-2.45070	-0.28641
100	C	C59	13.73402	-0.40090	-0.05964
101	H	H14	14.14756	-1.41731	-0.16668
102	C	C60	14.70168	0.56669	0.07403

Chart 1. Structural coordinates of the E isomer of $ZnTPPF_{20}CN$

$ZnTPPF_{20}CN\_Z$

Structure parameters

#	Element	Atom	x	y	z
1	N	N1	−1.32534	0.64116	0.11766
2	N	N2	−3.87647	2.02702	−0.03395
3	N	N3	−5.26263	−0.50728	−0.14624
4	N	N4	−2.72282	−1.88732	−0.02664
5	C	C1	−0.22584	−0.18145	0.13875
6	C	C2	1.00015	0.62530	0.08475
7	C	C3	0.57894	1.94621	0.05935
8	C	C4	−0.85407	1.93778	0.08593
9	C	C5	−1.64791	3.10818	0.06557
10	C	C6	−3.05480	3.13327	0.01811
11	C	C7	−3.84869	4.34522	0.01697
12	C	C8	−5.15750	3.95834	−0.05651
13	C	C9	−5.16624	2.50918	−0.07978
14	C	C10	−6.33856	1.72564	−0.15651
15	C	C11	−6.36647	0.31522	−0.19293
16	C	C12	−7.57783	−0.47818	−0.26219
17	C	C13	−7.19057	−1.78900	−0.26944
18	C	C14	−5.74375	−1.79772	−0.18177
19	C	C15	−4.95608	−2.96676	−0.12594
20	C	C16	−3.54709	−2.99109	−0.03320
21	C	C17	−2.76380	−4.19986	0.12474
22	C	C18	−1.45615	−3.81276	0.22685
23	C	C19	−1.44043	−2.36759	0.12753
24	C	C20	−0.26701	−1.58983	0.20191
25	H	H1	1.22147	2.82691	−0.01419
26	H	H2	−3.45389	5.36263	0.07444
27	H	H3	−6.04208	4.59821	−0.09867
28	H	H4	−8.59610	−0.08235	−0.29178
29	H	H5	−7.82854	−2.67419	−0.33453
30	H	H6	−3.16844	−5.21373	0.18040
31	H	H7	−0.57761	−4.44551	0.37961
32	C	C21	−7.64465	2.44175	−0.19895
33	C	C22	−8.06092	3.27970	0.85401
34	C	C23	−8.52270	2.30917	−1.29214
35	C	C24	−9.28765	3.95713	0.82797
36	C	C25	−9.75896	2.96796	−1.34079
37	C	C26	−10.14154	3.79733	−0.27477
38	C	C27	−0.93407	4.41436	0.09809
39	C	C28	−1.05077	5.34732	−0.95039
40	C	C29	−0.10998	4.77208	1.18317
41	C	C30	−0.38925	6.58289	−0.92482
42	C	C31	0.56633	5.99880	1.23204
43	C	C32	0.42342	6.90802	0.17238
44	C	C33	1.01856	−2.31271	0.40554
45	C	C34	1.57574	−3.13707	−0.59001
46	C	C35	1.72949	−2.17141	1.61067
47	C	C36	2.80473	−3.77847	−0.40305
48	C	C37	2.96116	−2.80506	1.82310
49	C	C38	3.49824	−3.60405	0.80434
50	C	C39	−5.66026	−4.27923	−0.12901
51	C	C40	−5.42360	−5.23754	−1.13366
52	C	C41	−6.57156	−4.62993	0.88613
53	C	C42	−6.05471	−6.48910	−1.13295
54	C	C43	−7.22042	−5.87223	0.90856
55	C	C44	−6.95740	−6.80625	−0.10569
56	Zn	Zn1	−3.30312	0.06964	−0.03116
57	F	F1	−1.82169	5.06148	−2.01417
58	F	F2	−0.52125	7.44792	−1.93784
59	F	F3	1.06004	8.08293	0.20845
60	F	F4	1.33839	6.31058	2.28056
61	F	F5	0.04315	3.92074	2.21208
62	F	F6	−8.18189	1.52206	−2.32838
63	F	F7	−10.56877	2.81747	−2.39490
64	F	F8	−11.31517	4.43385	−0.30992
65	F	F9	−7.26367	3.45146	1.92426
66	F	F10	−9.65077	4.74654	1.84593
67	F	F11	−4.55941	−4.96176	−2.12628
68	F	F12	−5.80561	−7.37873	−2.10189
69	F	F13	−7.56476	−7.99661	−0.09276
70	F	F14	−8.07631	−6.17568	1.89161
71	F	F15	−6.83713	−3.75779	1.87454
72	F	F16	1.23606	−1.38367	2.58028
73	F	F17	3.62716	−2.64317	2.97343
74	F	F18	4.69465	−4.18769	0.96385
75	F	F19	3.35435	−4.52817	−1.37258
76	F	F20	0.92432	−3.30494	−1.75337
77	C	C45	2.35157	0.23527	0.00321
78	C	C46	3.56963	0.06008	−0.08444
79	C	C47	4.97797	−0.03323	−0.11176
80	C	C48	5.78976	0.97778	0.42367
81	C	C49	5.66836	−1.16471	−0.66639
82	C	C50	7.19851	0.89835	0.41666
83	H	H8	5.30092	1.85123	0.87443
84	C	C51	7.11904	−1.25388	−0.64586
85	C	C52	7.92000	−0.18096	−0.11435
86	H	H9	7.75175	1.72223	0.88665
87	N	N5	5.06620	−2.22612	−1.23574
88	N	N6	7.57436	−2.40943	−1.18534
89	S	S1	6.26001	−3.24948	−1.65361
90	C	C53	9.39139	−0.18839	−0.12316
91	C	C54	10.11547	1.03110	−0.07936
92	C	C55	10.14104	−1.38959	−0.18212
93	C	C56	11.50480	1.06511	−0.06098
94	H	H10	9.56977	1.98444	−0.09820
95	C	C57	11.53195	−1.35593	−0.16514
96	H	H11	9.61140	−2.34768	−0.25638
97	C	C58	12.25909	−0.13589	−0.09233
98	H	H12	12.03385	2.02269	−0.03409
99	H	H13	12.08894	−2.30330	−0.20392
100	H	H14	14.02383	−1.30488	−0.25076
101	C	C59	13.70615	−0.26021	−0.09431
102	C	C60	14.79335	0.57750	0.05819
103	C	C61	16.08276	−0.05867	−0.03747
104	N	N7	17.10508	−0.62865	−0.13471
105	C	C62	14.95204	2.02862	0.34419
106	O	O1	16.04617	2.53705	0.51783
107	O	O2	13.79783	2.75891	0.40731
108	H	H15	14.10270	3.66853	0.61436

Chart 2. Structural coordinates of the Z isomer of $ZnTPPF_{20}CN$

Synthesis of $ZnTPPF_{20}CN$

We used reagent-grade purity chemicals purchased from Merck, with the exception of free-base 5,10,15,20-tetrakis(pentafluorophenyl)porphyrin that was acquired from PorphyChem Sas. We employed a Milli-Q apparatus to obtain doubly distilled water. We characterized all synthetic intermediates and $ZnTPPF_{20}CN$ by ^{1}H- and ^{19}F-NMR spectroscopy, recording the spectra on a Bruker Avance DRX-400 spectrometer using $CDCl_3$ or THF-d_8 as solvent (Sigma-Aldrich).

We prepared $ZnTPPF_{20}CN$ according to Scheme 1.

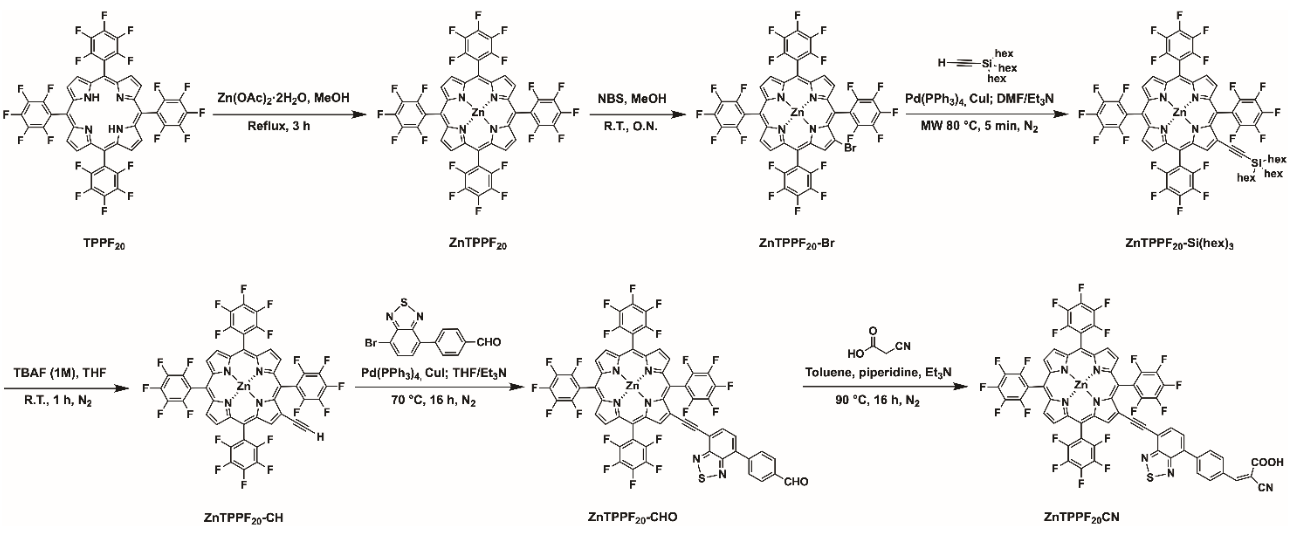

Scheme S1. Synthetic protocol to $ZnTPPF_{20}CN$

$ZnTPPF_{20}$

In a two-neck round-bottomed flask, 433 *mg* of $Zn(OAc)_2 \cdot 2H_2O$ (1.97 *mmol*, 4 equivalents) were added to a solution of 500 *mg* of $TPPF_{20}$ (0.51 *mmol*, 1 equivalent) in methanol (80 *mL*), and the mixture was refluxed for 3h, then cooled to RT, and the solvent evaporated *in vacuo*. The crude was washed with purified water (30 *mL*), then dried over a Buchner filter, affording $ZnTPPF_{20}$ as a purple powder in almost quantitative yield.

^{1}H-NMR (400 *MHz*, $CDCl_3$, 25 *°C*) δ, *ppm*: 9.03 (8H, s)

^{19}F-NMR (377 *MHz*, $CDCl_3$, 25 *°C*) δ, *ppm*: -136.73 (8F, m), -151.75 (4F, m), -161.9 (8F, m)

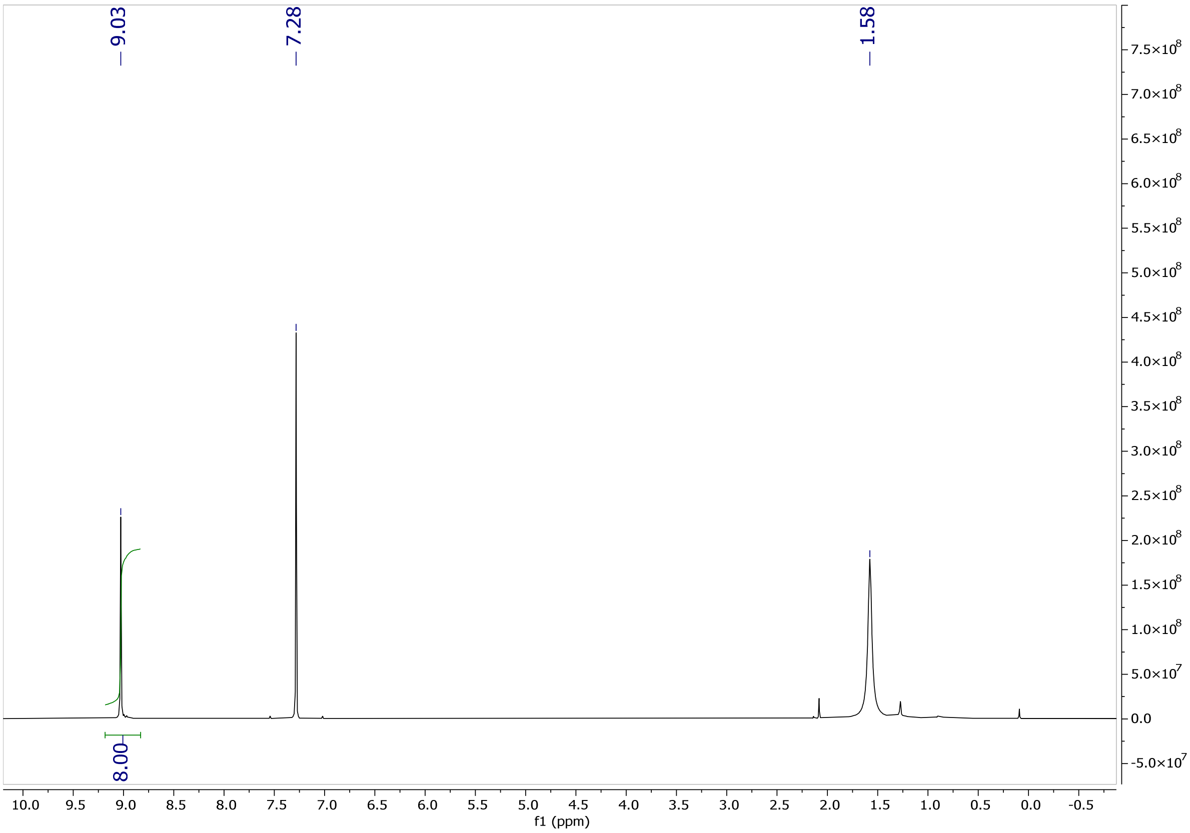

Figure S6. ^{1}H-NMR of $ZnTPPF_{20}$ in $CDCl_3$

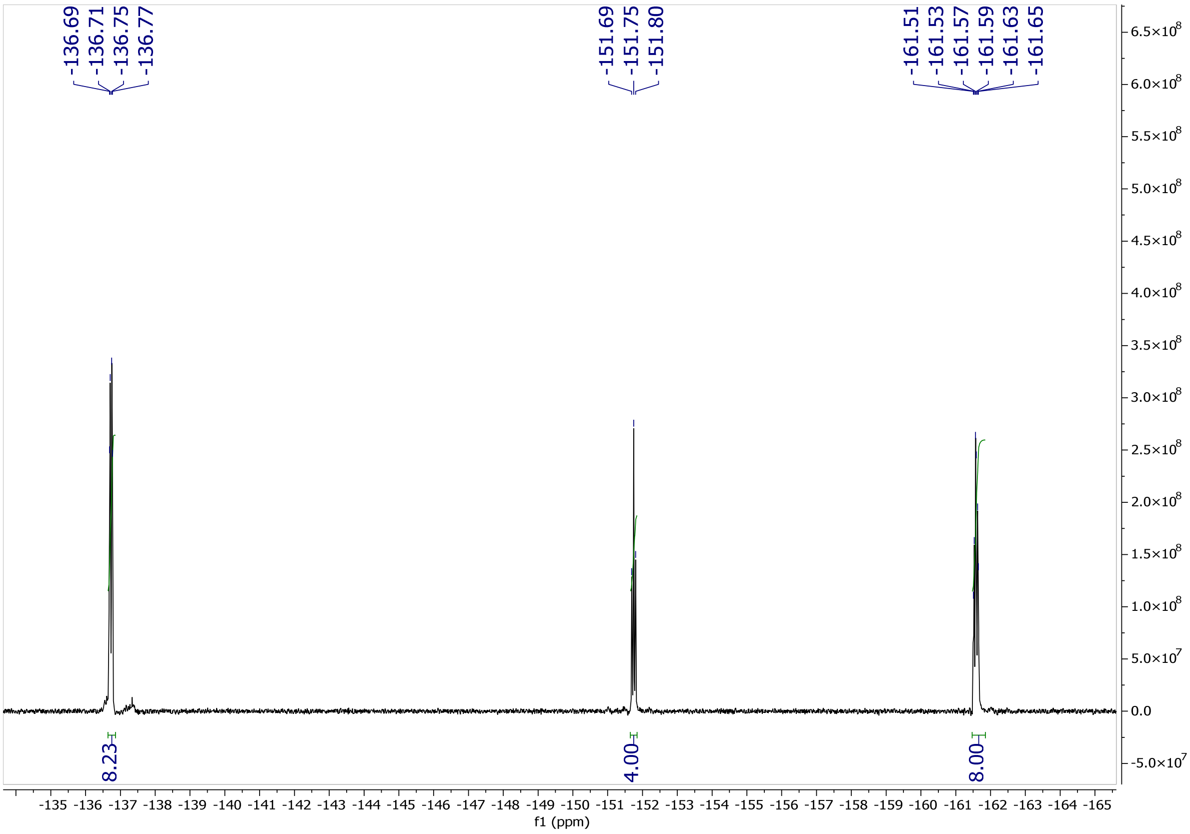

Figure S7. ^{19}F-NMR of $ZnTPPF_{20}$ in $CDCl_3$

ZnTPPF$_{20}$Br

In a round bottomed flask, 100 *mg* (0.096 *mmol*, 1 equivalent) of ZnTPPF$_{20}$ were dissolved in 50 *mL* of methanol. 17.1 *mg* (0.096 *mmol*, 1 equivalent) of NBS were added to the solution, which was left at room temperature and under vigorous stirring overnight. The crude was retrieved evaporating the methanol *in vacuo*, then it was dissolved in dichloromethane and washed three times with water (3x10*mL*), the organic phase was dried over Na_2SO_4 and the solvent evaporated *in vacuo*. The crude was purified by filtration on a silica plug, using Dichloromethane/ n-hexane = 3/7 as eluent. The process afforded a mixture of ZnTPPF$_{20}$ and ZnTPPF$_{20}$-Br as a purple powder (99.7 *mg*, 75% yield of ZnTPPF$_{20}$-Br determined by ^{1}H-NMR).

^{1}H-NMR (400 *MHz*, $CDCl_3$, 25 *°C*) *δ*, *ppm*: 9.17 (1H, d, J = 12), 9.06 (6H, m)

^{19}F-NMR (377 *MHz*, $CDCl_3$, 25 *°C*) *δ*, *ppm*: -136.67 (6F, m), -137.07 (2F, m), -151.79 (4F, m), -161.50 (6F, m), -162.67 (2F, m)

ESI-ITMS: *m/z* calculated for $C_{44}H_7BrF_{20}N_4Zn$ = 1116.07; found = 1115.27 [M – 1]

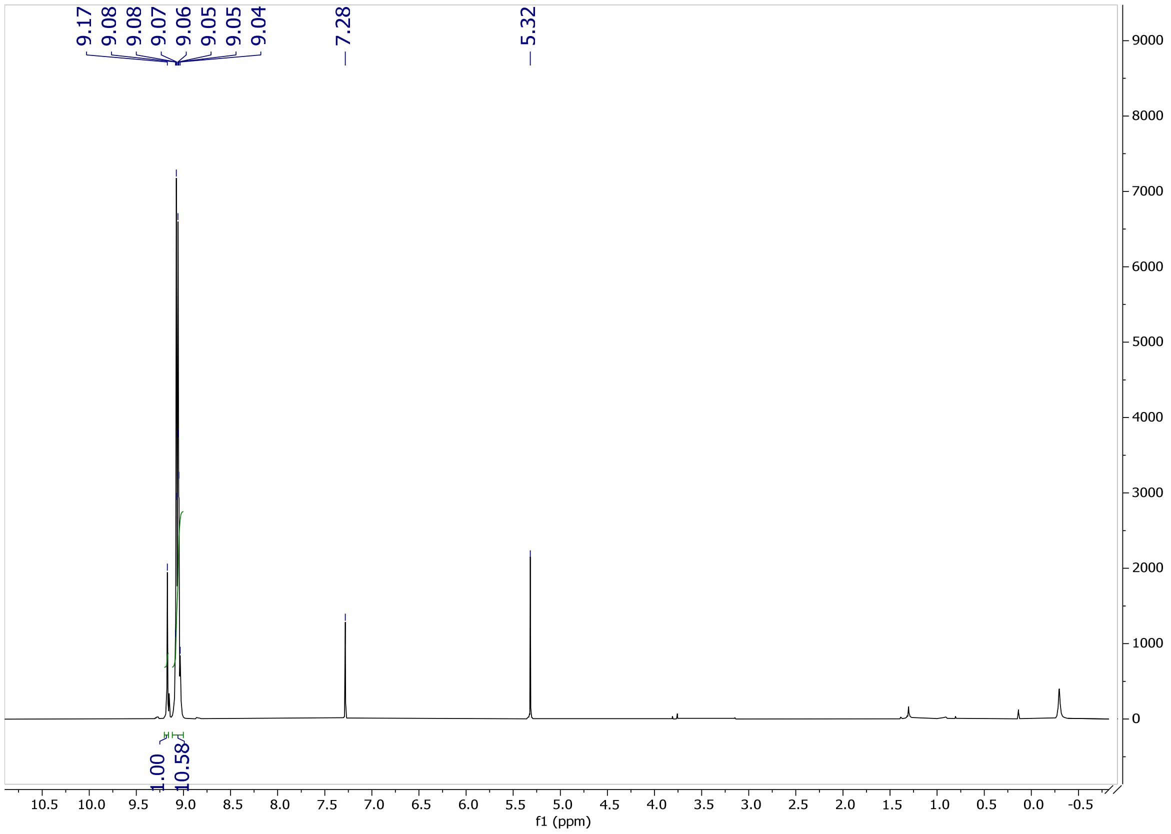

Figure S8. ^{1}H-NMR of ZnTPPF$_{20}$Br in $CDCl_3$

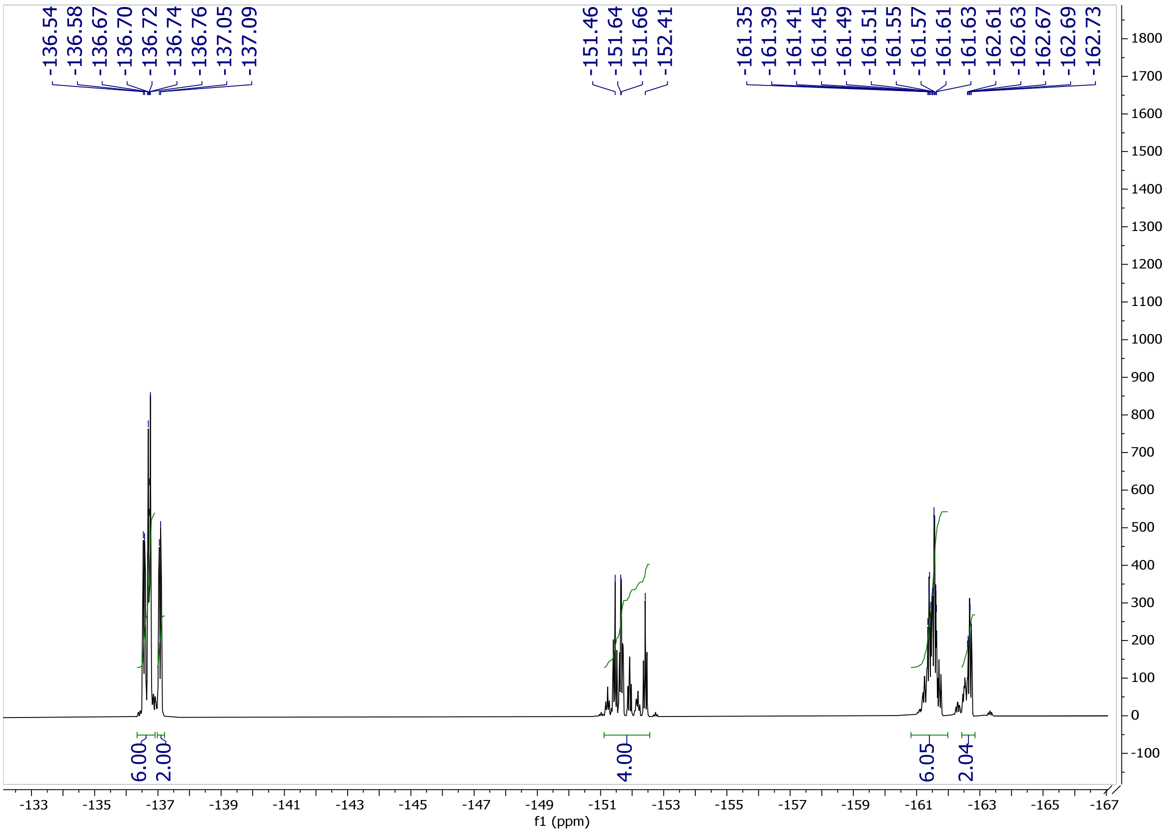

Figure S9. ^{19}F-NMR of $ZnTPPF_{20}Br$ in $CDCl_3$

$ZnTPPF_{20}Si(hex)_3$

In a microwave reaction vial equipped with a stirring bar, 50 *mg* of $ZnTPPF_{20}$-Br (0.04 *mmol*, 1 equivalent), 25 *mg* of ethynyltrihexylsilane (0.08 *mmol*, 2 equivalents) and 9.24 *mg* of palladium tetrakis (0.008 *mmol*, 0.2 equivalents) were dissolved in 1.5 *mL* of freshly distilled triethylamine and 2 *mL* of dry dimethylformamide. The resulting mixture was bubbled for 10 min with gaseous nitrogen to remove O_2, then 0.8 *mg* of CuI (0.004 *mmol*, 0.1 equivalents) were added. The vial was sealed, and the mixture was allowed to react in the microwave cavity for at 80°C for 5 minutes. The solvents were removed under *vacuum,* the crude dissolved in 20 *mL* of dichloromethane and washed with water (3x10 *mL*). The collected organic phases were dried over Na_2SO_4 and the solvent evaporated. The solid was filtrated through a silica plug, first with 100% hexane to remove unreacted silane, then with 100% dichloromethane to collect porphyrin fraction. Finally, the pure $ZnTPPF_{20}$-Si(hex)$_3$ was obtained as a purple amorphous solid (33.6 *mg*, 62% yield) by flash chromatography (from 8/2 n-hexane/dichloromethane to 100% dichloromethane).

^{1}H-NMR (400 *MHz*, $CDCl_3$, 25 *°C*) *δ*, *ppm*: 9.17 (1H, s), 8.98 (6H, m), 1.45 (24H, m), 0.94 (9H, m), 0.83 (6H, m)

^{19}F-NMR (377 *MHz*, $CDCl_3$, 25 *°C*) *δ*, *ppm*: -136.56 (8F, m), -152.48 (4F, m), -161.56 (8F, m)

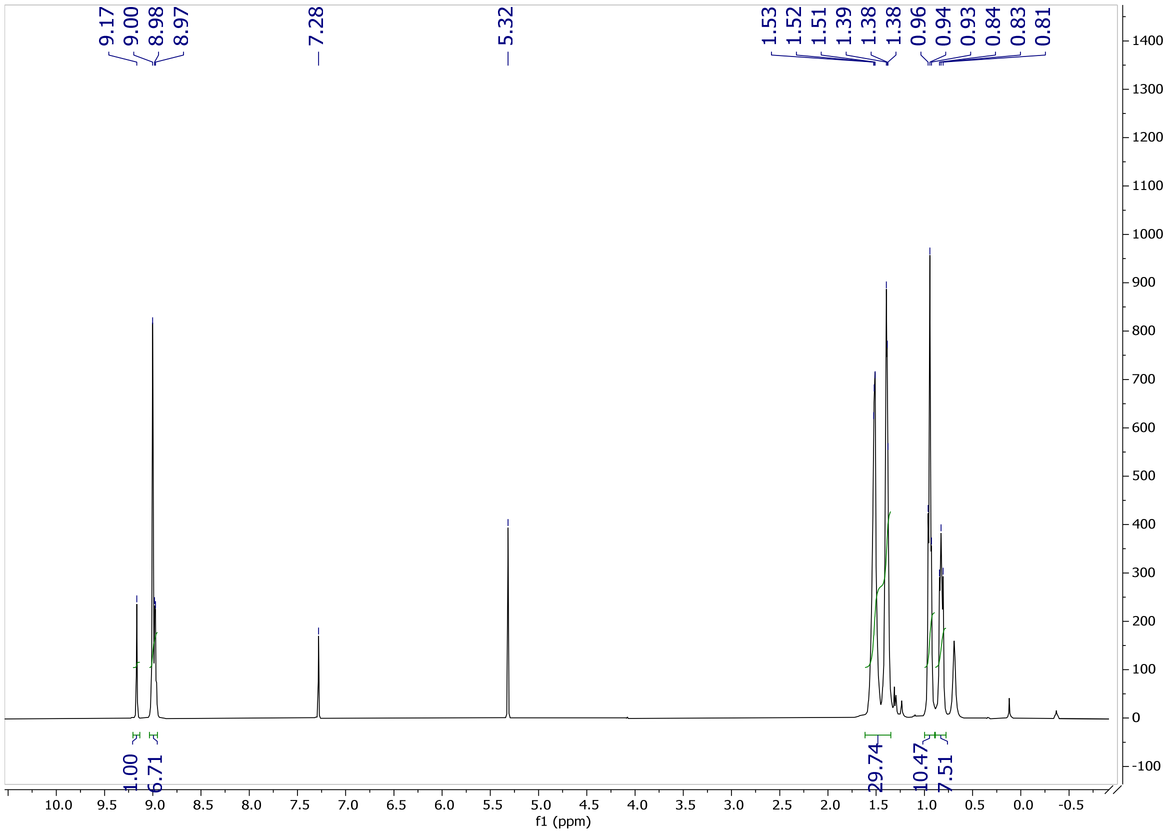

Figure S10. ^{1}H-NMR of $ZnTPPF_{20}Si(hex)_3$ in $CDCl_3$

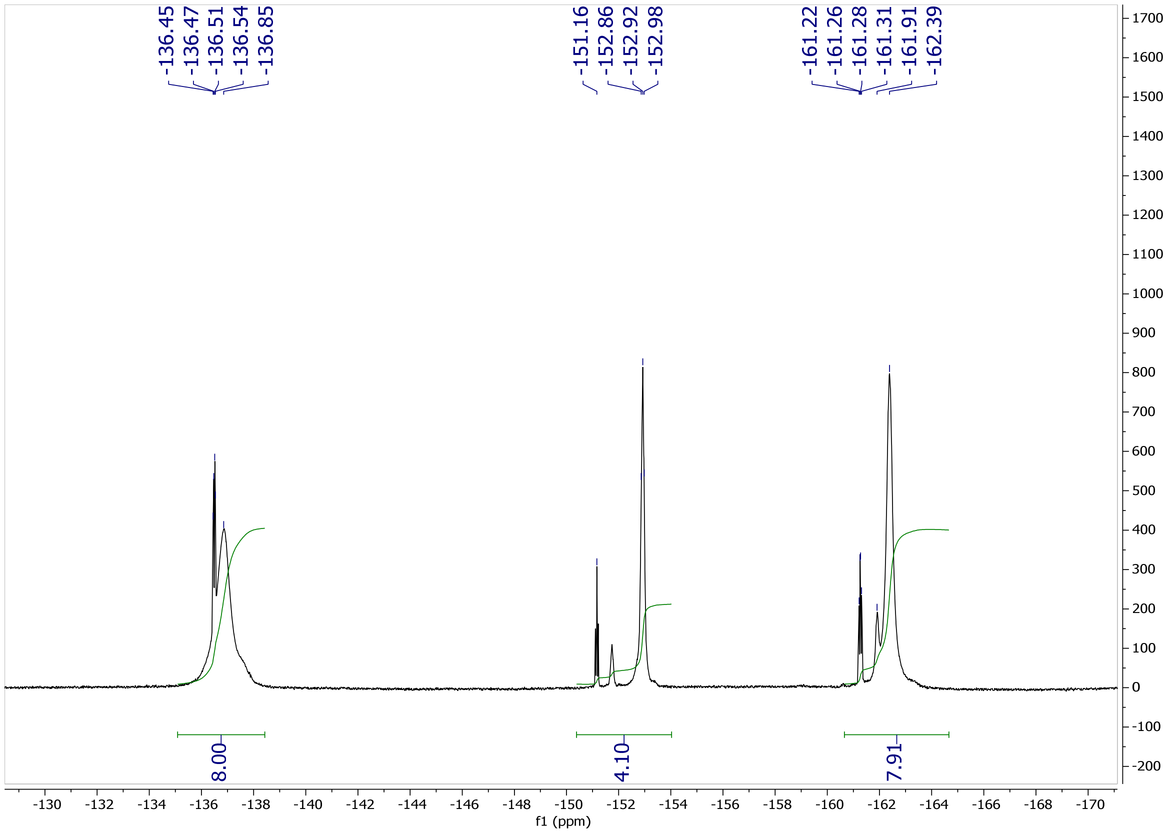

Figure S11. ^{19}F-NMR of $ZnTPPF_{20}Si(hex)_3$ in $CDCl_3$

ZnTPPF₂₀CH

In a dry Schlenk tube, under nitrogen flow, TBAF 0.150 *mL* (solution 1 M in tetrahydrofuran; 0.055 *mmol*, 2 equivalents) were added dropwise under stirring to a solution of 150 *mg* (0.111 *mmol*, 1 equivalent) of $ZnTPPF_{20}$-Si(hex)$_3$ in 5 *mL* of tetrahydrofouran. The mixture was stirred for 1 hour at RT, then the reaction was quenched with H_2O. The crude product was washed with water (3x10 *mL*) and dichlorometane (3x15 *mL*), the organic phase was dried over Na_2SO_4 and the solvent evaporated *in vacuo*. The crude was purified by gravimetric chromatography (eluent: from n-hexane 100% to dichloromethane 100%). Affording $ZnTPPF_{20}$-CH as a purple powder (116.9 *mg,* 99% yield).

^{1}H-NMR (400 *MHz*, $CDCl_3$, 25 *°C*) *δ*, *ppm*: 9.27 (1H, s), 9.01(6H, m), 3.70 (1H, s)

^{19}F-NMR (377 *MHz*, $CDCl_3$, 25 *°C*) *δ*, *ppm*: -136.85 (8F, m), -151.99 (3F, m), -154.03 (1F, s), -161.92 (6F, s), -163.77 (2F, s)

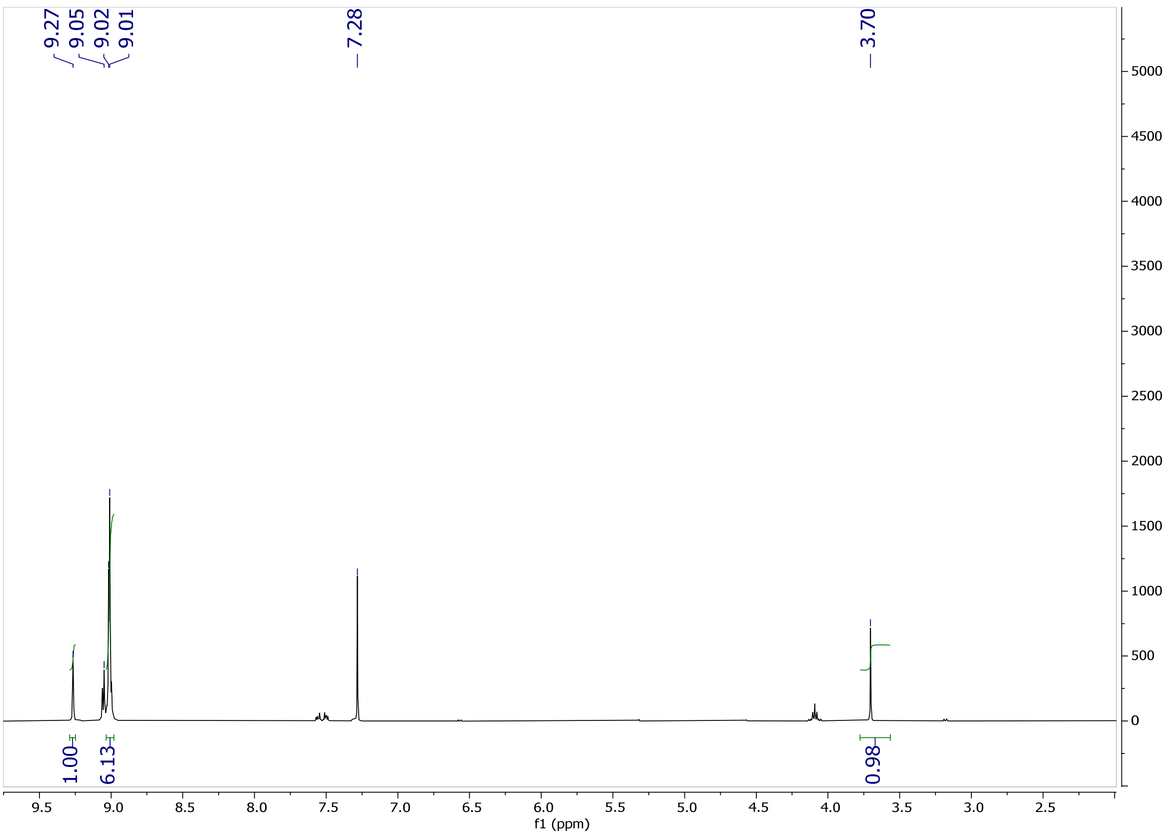

Figure S12. ^{1}H-NMR of $ZnTPPF_{20}$CH in $CDCl_3$

ZnTPPF₂₀CHO

In a dry Schlenk tube, under nitrogen flow, 120 *mg* of $ZnTPPF_{20}$-CH (0.113 *mmol*, 1 equivalent), 72 *mg* of 4-(7-bromobenzo[1,2,5]thiadiazol-4-yl)benzaldehyde (0.226 *mmol*, 2 equivalents) and 20 *mg* of palladium tetrakis (0.017 *mmol*, 0.15 equivalents) were dissolved in 7 *mL* of freshly distilled

triethylamine and 7 *mL* of dry tetrahydrofuran. After bubbling gaseous N_2 into the solution for 10 min i to remove O_2 traces from the solvents, CuI 2 *mg* (0.011 *mmol*, 0.1 equivalents) were added. Then the mixture was kept at 70°C under vigorous stirring for 16 hours, under a nitrogen atmosphere. After the evaporation of the solvent in vacuo, the mixture in dissolved in 20 *mL* of Dichloromethane and washed with H_2O (3x10 *mL*). The organic phase was dried over Na_2SO_4 and the solvent evaporated. The crude was purified by gravimetric chromatography (eluent: from 100% n-hexane to 100% Dichloromethane). Pure ZnTPPF$_{20}$-CHO was collected as a purple-greenish powder (123 *mg*, 83% yield).

^{1}H-NMR (400 *MHz*, $CDCl_3$, 25 *°C*) *δ*, *ppm*: 10.14 (1H, s), 9.39 (1H, s), 9.00 (6H, m), 8.29 (2H, D, J = 8.1 *Hz*), 8.12 (2H, d, J = 8.1 *Hz*), 8.01 (1H, d, J = 7.3 *Hz*), 7.94 (1H, *d*, J = 7.3 *Hz*)

^{19}F-NMR (377 *MHz*, $CDCl_3$, 25 *°C*) *δ*, *ppm*: -136.75 (8F, broad s), -151.81 (3F, m), -154.02 (1F, m), -162.37 (6F, broad s), -163.32 (2F, s)

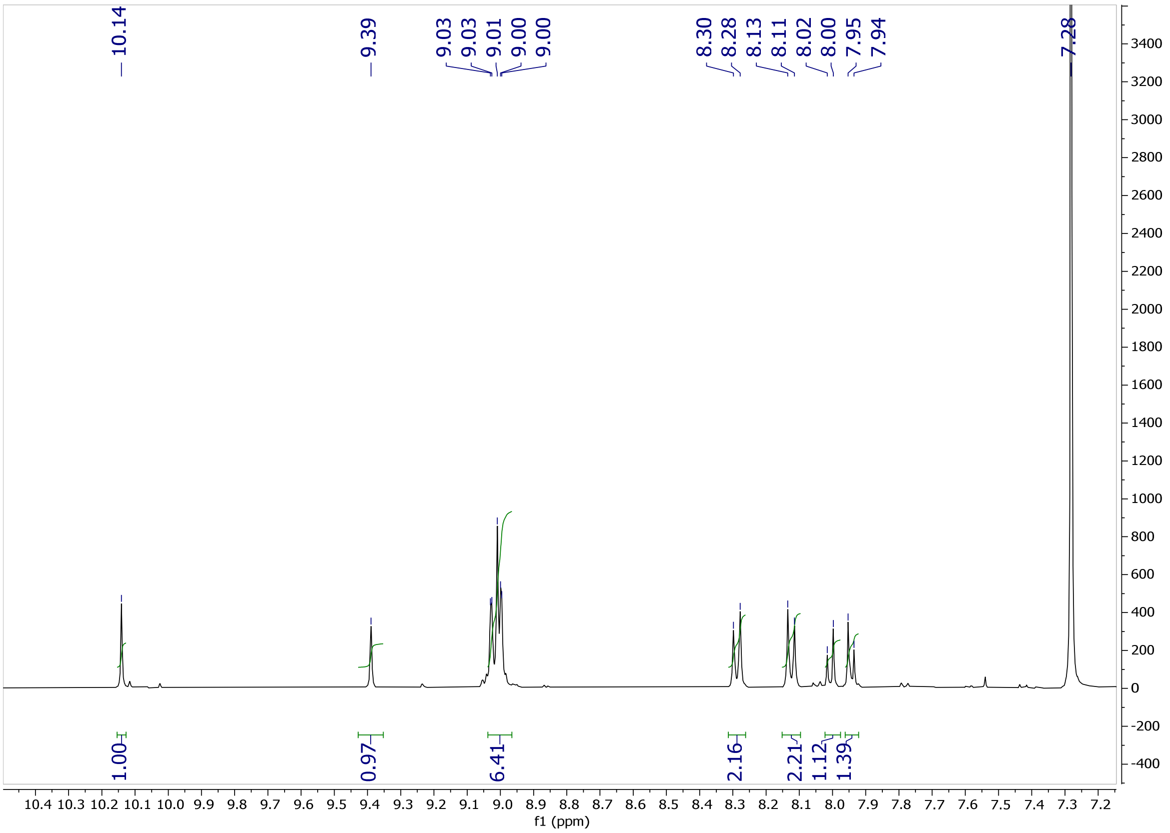

Figure S13. ^{1}H-NMR of ZnTPPF$_{20}$CHO in $CDCl_3$

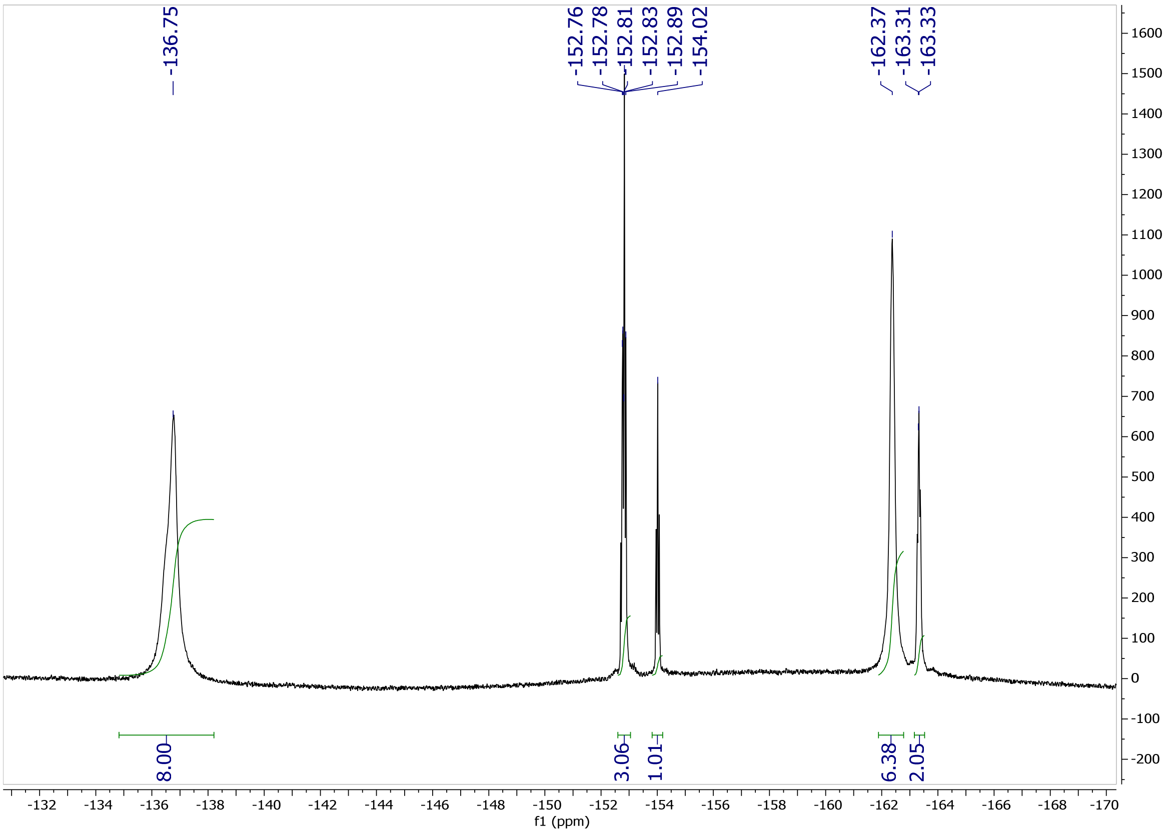

Figure S14. ^{19}F-NMR of $ZnTPPF_{20}CHO$ in $CDCl_3$

$ZnTPPF_{20}CN$

In a dry Schlenk tube, under nitrogen flow, 44 *mg* of $ZnTPPF_{20}$-CHO (0.033 *mmol*, 1 equivalent) were dissolved into 1 *mL* of toluene. 14.4 *mg* of cyanoacetic acid (0.169 *mmol*, 5 equivalents) was added under stirring. After the addition of 34 *µL* of piperidine (0.33 *mmol*, 10 equivalents) and 6 *µL* of triethylamine (0.33 *mmol*, 10 equivalents) the solution was heated at 90°C for 16h. After cooling down to RT the mixture was quenched with 5 *mL* of H_3PO_4 2 *M* in water and washed with dichloromethane. The organic layer was dried over Na_2SO_4 and the solvents evaporated *in vacuo*. The crude was then purified by filtration on a silica plug starting with 100% dichloromethane to remove the impurities, and using then dichloromethane /methanol = 9/1 to collect $ZnTPPF_{20}$-CN as a purple- greenish amorphous solid (33.5 *mg*, 73% yield).

^{1}H-NMR (400 *MHz*, THF-d_8 with 1 drop of D_2O, 25 *°C*) *δ*, *ppm*: 9.54 (1H, s), 9.11 (6H, m), 8.40 (2H + 1H, A***B***, J = 8 *Hz*), 8.29 (2H, ***A***B, J = 8 *Hz*), 8.19 (1H, A***B***, J = 8 *Hz*), 8.10 (1H, ***A***B, J = 8 *Hz*)

^{19}F-NMR (377 *MHz*, THF-d_8 with 1 drop of D_2O, 25 *°C*) *δ*, *ppm*: -136.62 (2F, d, J = 23 *Hz*),), -136.88 (6F, t, *J* = 30.1 Hz), -151.80 (3F, m), 152.97 (1F, m), -161.63 (6F, m), -162.58 (2F, m)

ESI-ITMS: *m/z* calculated for $C_{62}H_{15}F_{20}N_7O_2SZn$ = 1367.27; found = 1366.72 [M – 1].

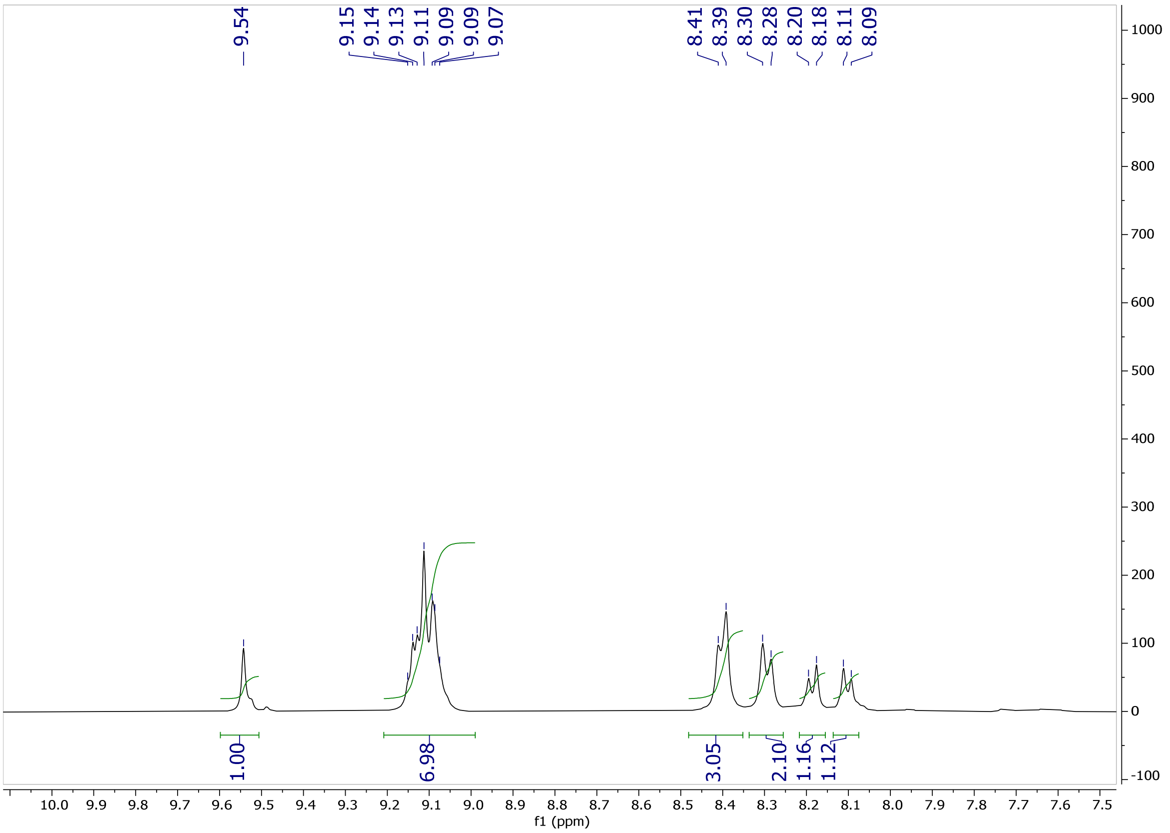

Figure S15. ^{1}H-NMR of $ZnTPPF_{20}CN$ in THF-d_8 with 1 drop of D_2O

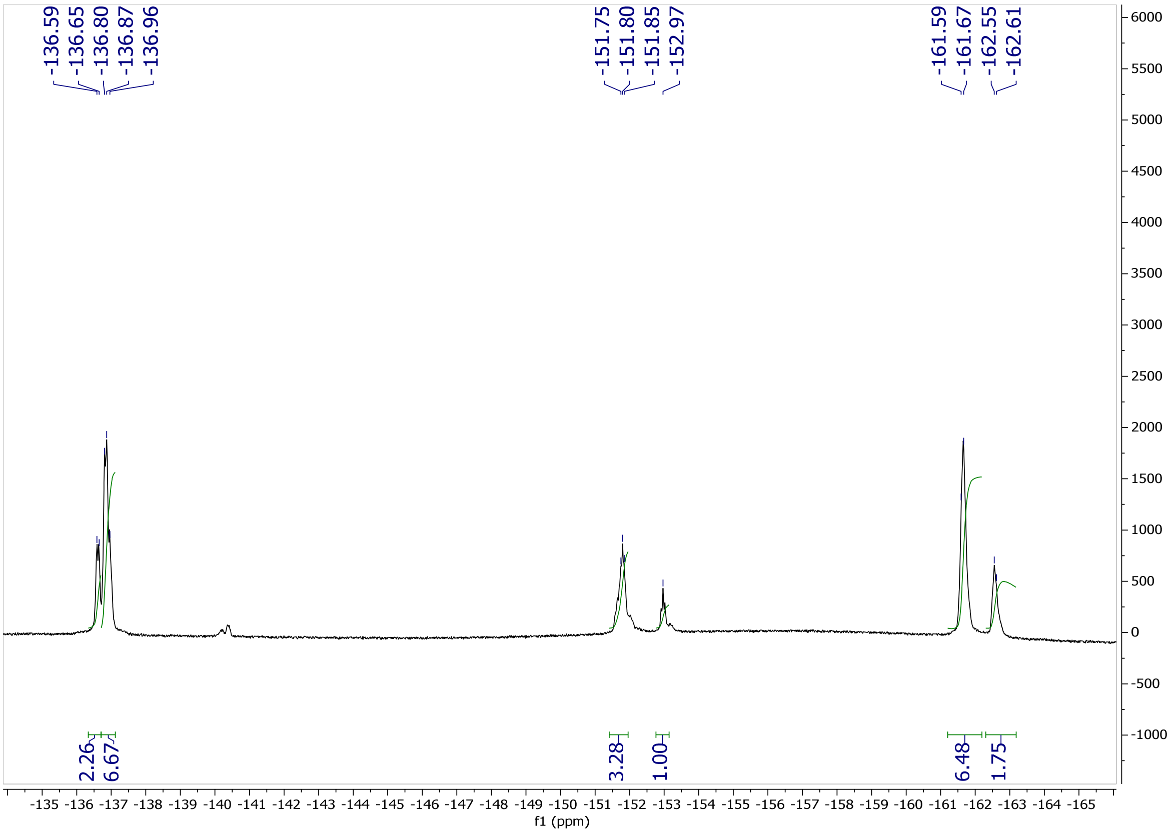

Figure S16. ^{19}F-NMR of $ZnTPPF_{20}CN$ in THF-d_8 with 1 drop of D_2O

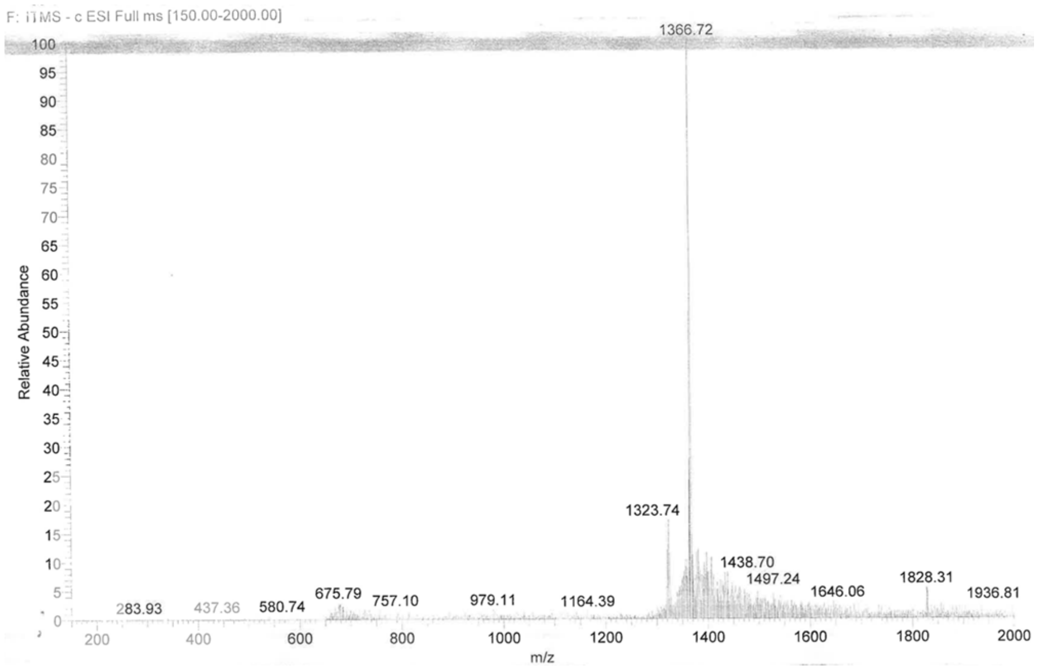

Figure S17. ESI-ITMS spectrum of $ZnTPPF_{20}CN$

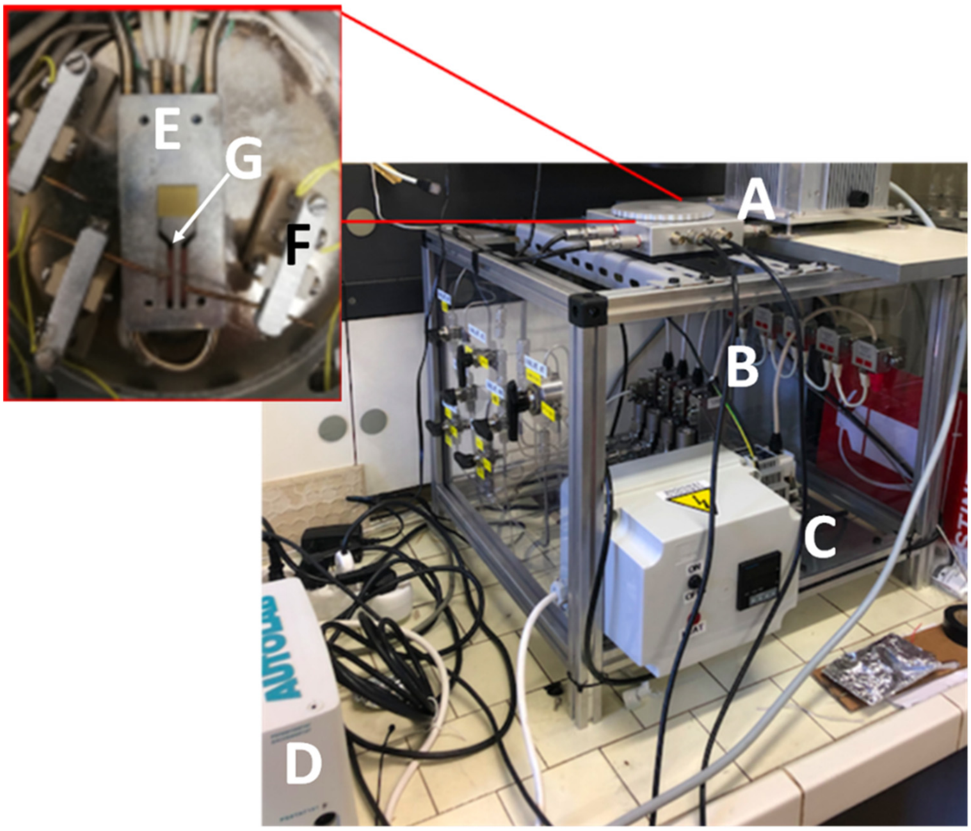

Figure S18. Image of the gas sensor testing setup: A) homemade stainless steel in-situ sensor testing cell; B) gas manifold; C) cell temperature controller; D) Autolab potentiostat. Inset: inside of the in-situ sensor testing cell: E) heating plate for temperature control; F) needle-electrical connectors; G) Pt-interdigitated electrode covered by the synthesized sensing materials.
